# Alcohol-induced gut microbiome dysbiosis enhances the colonization of *Klebsiella pneumoniae* on the mouse intestinal tract

**DOI:** 10.1128/msystems.00052-24

**Published:** 2024-02-12

**Authors:** Mengke Shen, Huajie Zhao, Meiqing Han, Lin Su, Xiaojian Cui, Duan Li, Liang Liu, Chuansheng Wang, Fan Yang

**Affiliations:** 1 Department of Pathogenic Biology, School of Basic Medical Science, Xinxiang Medical University, Xinxiang, China; 2 Department of Pathogenic Biology and Immunology, Sanquan College of Xinxiang Medical University, Xinxiang, China; 3 The Second Aﬃliated Hospital of Xinxiang Medical University, Henan Key Laboratory of Biological Psychiatry, Xinxiang Medical University, Xinxiang, China; University of California San Diego, La Jolla, California, USA

**Keywords:** alcohol consumption, gut microbiome dysbiosis, *Klebsiella pneumoniae*, colonization, lithocholic acid

## Abstract

Chronic alcohol consumption, an important risk factor for diseases and deaths, can cause intestinal microbiota dysbiosis and increase the infection of some opportunistic pathogens. However, the current studies on the effects of alcohol-induced intestinal microbiota dysbiosis on gut colonization of *Klebsiella pneumoniae* are still scarce. In the present study, we established a binge-on-chronic alcohol model in mice to identify the characteristics of alcohol-induced intestinal microbiome and metabolite dysbiosis using multi-omics and explored the effects and potential mechanisms of these dysbioses on the intestinal colonization of *K. pneumoniae*. The results show that chronic alcohol consumption alters the diversity and composition of gut microbiota (including bacteria and fungi), decreases the complexity of the interaction between intestinal bacteria and fungi, disturbs the gut metabolites, and promotes the colonization of *K. pneumoniae* on the gut of mice. The relevance analyses find that alcohol-induced gut microbiome dysbiosis has a strong correlation with the alteration of secondary bile acids. *In vitro* results suggest that the high concentration of lithocholic acid, a secondary bile acid, could significantly inhibit the proliferation of *K. pneumoniae*, and the adhesion of *K. pneumoniae* to Caco-2 cells. Our results indicate that alcohol-induced microbiome dysbiosis contributes to decreased levels of secondary bile acids, which was one of the main reasons affecting the colonization of *K. pneumoniae* in mice’s intestines. Some secondary bile acids (e.g., lithocholic acid) might be a potential drug to prevent the colonization and spread of *K. pneumoniae*.

**IMPORTANCE:**

Alcohol is one of the most commonly misused substances in our lives. However, long-term heavy drinking will increase the colonization of some opportunistic pathogens (e.g., *Klebsiella pneumoniae*) in the body. Here, we revealed that binge-on-chronic alcohol consumption disrupted the balance between gut bacteria and fungi, induced the gut microbiome and metabolites dysbiosis, and promoted the colonization of *K. pneumoniae* in the intestine of mice. In particular, alcohol-taking disrupted intestinal bile acid metabolism and reduced the lithocholic acid concentration. However, a high concentration of lithocholic acid can protect against intestinal colonization of *K. pneumoniae* by inhabiting the bacterial growth and adhesion to the host cell. Hence, regulating the balance of gut microbiota and intestinal bile acid metabolism may be a potential strategy for reducing the risk of *K. pneumoniae* infection and spread.

## INTRODUCTION

Alcohol consumption has been recognized as a significant risk factor for chronic disease and injury (1). Continued excessive alcohol consumption is likely to damage the nervous system (2, 3), heart and cardiovascular system (4, 5), digestive system (6, 7), respiratory system (8, 9), and many other organs. Chronic alcohol intake also impairs cognition, emotion, and behavior as well as increases the risk of depression (10, 11). In addition, alcohol consumption constitutes an independent risk factor for community-acquired pneumonia, and there is a dose-response relationship between them (8). Alcohol consumption affects the human immune system, resulting in increased susceptibility to infections such as bacterial pneumonia (9). Compared with the general population, alcoholics are more likely to be infected with *Streptococcus pneumoniae* (12, 13), *Klebsiella pneumoniae* (14)*, Mycobacterium tuberculosis* (15, 16), and *Helicobacter pylori* (17, 18)*,* and the severity of infection is much higher than that of the general population.

Alcohol consumption can cause systemic inflammatory changes and increase the infection of pathogenic bacteria through two mechanisms mediated by the gastrointestinal tract (19). Alcohol changes the composition of intestinal microbiota, which leads to intestinal microbiota dysbiosis (20–22), and triggers the increase of endotoxin (23). In addition, alcohol also reduces the innate immune response of mucosa, destroys the intestinal barrier, causes high permeability of the intestine (24–26), and further makes endotoxin and inflammatory reaction molecules enter the systemic circulation (27).

Currently, studies of alcohol-induced intestinal microbiota disruption have mainly focused on bacteria, and there is still a lack of data on alcohol misuse and intestinal fungi. Some researchers reported that alcohol-associated gut dysbiosis impaired host defense capacity, and increased host respiratory susceptibility to *K. pneumoniae* (14). The capacity of intestinal colonization of *K. pneumoniae* is an important factor contributing to endogenous infections. However, until now, it has not been reported whether alcohol-induced intestinal microbiota affects the colonization of *K. pneumoniae* in the intestine. Hence, in the present study, we have established a binge-on-chronic alcohol model of mice in order to identify the characteristics of intestinal microbiota dysbiosis and its effect on intestinal colonization by *K. pneumoniae* and tried to explore the potential mechanisms of alcohol-induced intestinal microbiota disruption on intestinal colonization by *K. pneumoniae*.

## RESULTS

### Binge-on-chronic alcohol consumption increased the mice’s intestinal colonization of *K. pneumoniae*

**Fig 1:**
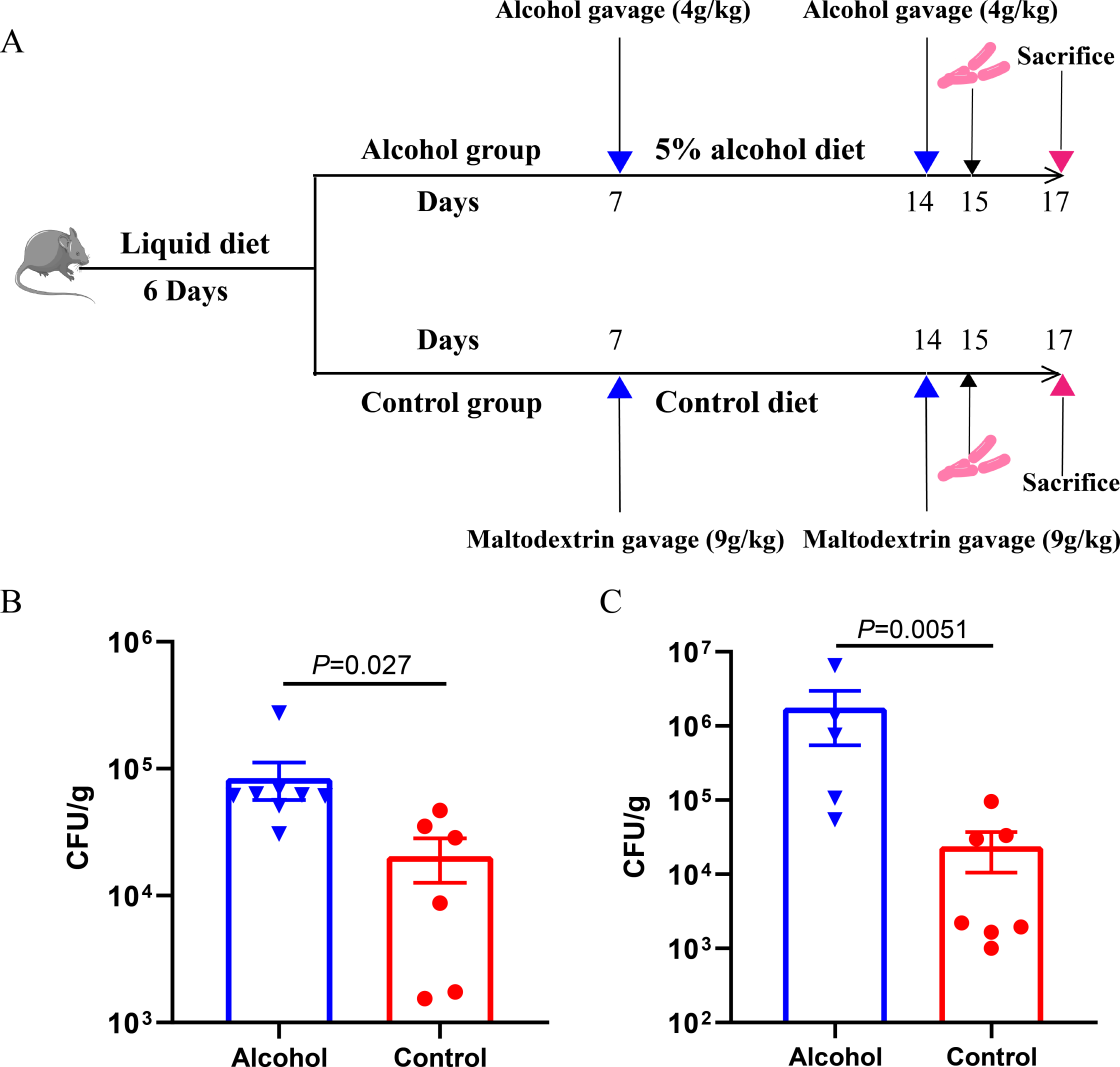
Binge-on-chronic alcohol consumption increased the mice’s intestinal colonization of *K. pneumoniae*. (**A**) Schematic diagram of binge-on-chronic alcohol consumption model. (**B**) The load of *K. pneumoniae* in fecal after infection at 24 h. (**C**) The load of *K. pneumoniae* in fecal at 48 h after infection. Data are represented as means ± SEM. *P < 0.05, **P < 0.01.

The experiment assessed the effect of alcohol consumption on the colonization of *K. pneumoniae* in mice’s intestinal tract. The binge-on-chronic alcohol model was performed according to Fig. 1A. The results showed that the intestinal colonization rates of *K. pneumoniae* in the alcohol group at 24 h (Fig. 1B) and 48 h (Fig. 1C) were significantly higher than those in the control group, which indicates that alcohol consumption increased the intestinal burden of *K. pneumoniae* in mice. Therefore, a high carrier rate may act as an important risk factor for endogenous infection of *K. pneumoniae* in clinical.

### Binge-on-chronic alcohol consumption altered the intestinal microbiota in mice

**Fig 2:**
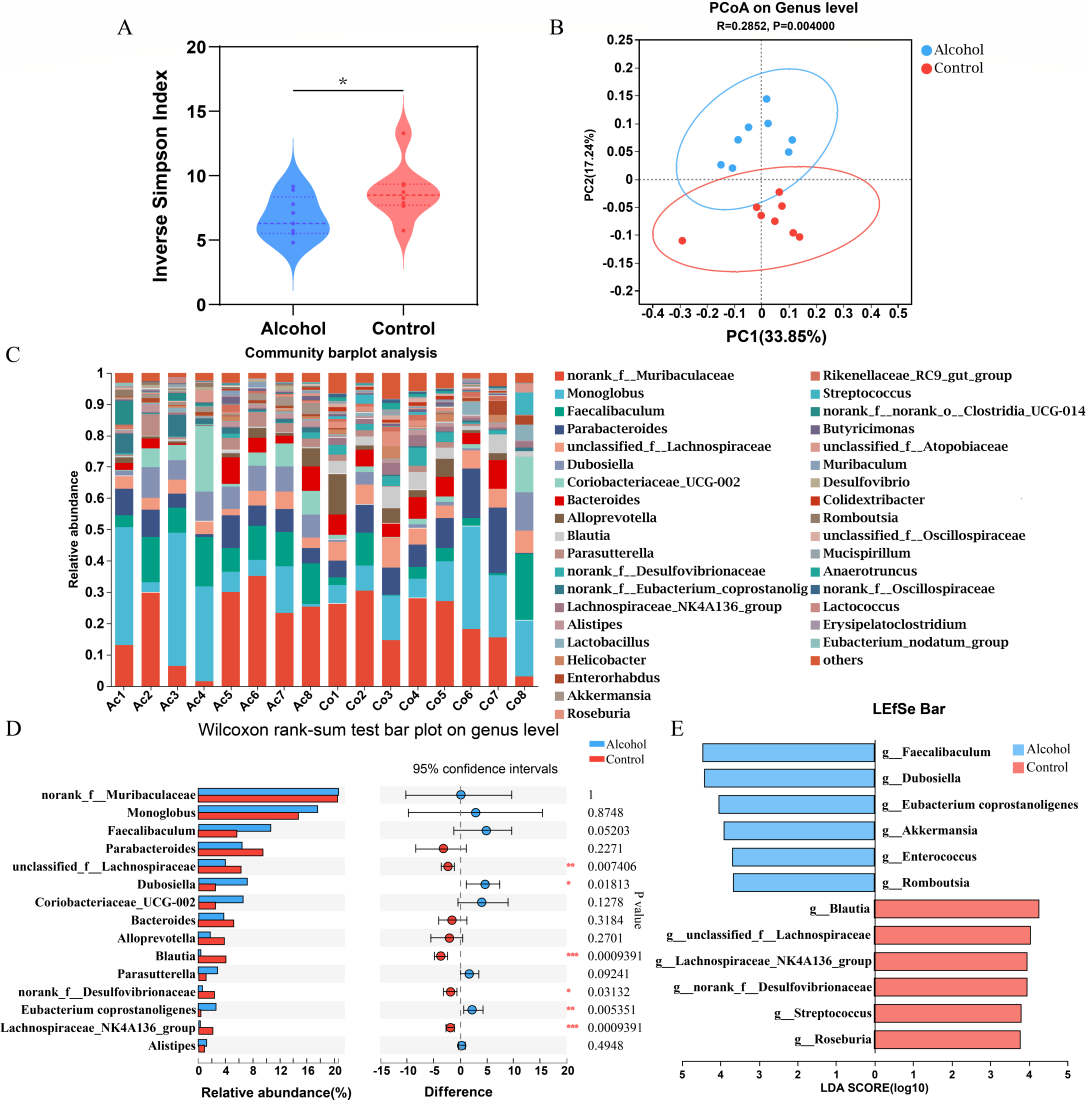
Binge-on-chronic alcohol consumption altered the intestinal microbiota. (**A**) The α-diversity of the microbiota at the genus level. (**B**) The PCoA is based on unweighted UniFrac distances between two groups. (**C**) The composition distribution of bacterial genera in all samples. (**D**) The difference in abundance of the top 15 bacterial genera between the two groups. (**E**) Linear discriminant analysis (LDA > 3.5).

We assessed the effect of alcohol intake on gut microbiota diversity using the inverse Simpson index. The result showed that the inverse Simpson index was significantly reduced in the alcohol group vs the control group (*P* < 0.05; Fig. 2A), which indicates that binge-on-chronic alcohol consumption decreased the community diversity of gut microbiota in mice. Based on unweighted UniFrac distances, principal coordinate analysis (PCoA) exhibited distinctly different clustering results of samples between the control group and the alcohol group (Fig. 2B), proving that binge-on-chronic alcohol consumption could cause alteration of microbiota community structure in mice. At the phylum level, *Firmicutes* and *Bacteroidetes* were the dominant phylum in the two groups, and there was no difference between the two groups (Table S1). At the genus level, 37 genera were obtained after removing genera with a relative abundance of less than 0.1%, and the alteration in these genera in alcohol and control groups is shown in Table S2. *Norank_f_ Muribaculaceae*, *Monoglobus*, *Parabacteroedes,* and *Faecalibaculum* were the dominant genera with a relative abundance of more than 5% in the two groups. The distribution of these genera in all samples is shown in Fig. 2C.

We selected bacterial genera with abundance in the top 15 for the Wilcoxon rank sum test. The results showed that six genera were significantly different between the control group and the alcohol group. Binge-on-chronic alcohol consumption decreased the relative abundances of *Lachnospiraceae_NK4A136_group*, *unclassified_f_Lachnospiraceae*, *norank_f_ Desulfovibrionaceae,* and *Blautia*, and markedly increased the relative abundances of *Dubosiella* and *norank_f_Eubacterium _coprostanoligenes-group* (Fig. 2D). The linear discriminant analysis effect size (LEfSe) analysis provided consistent results with Wilcoxon rank sum test (Fig. 2E). A cladogram for phylum to genus level abundance is shown in Fig. S1. Altogether, our data indicated that binge-on-chronic alcohol consumption induced intestinal microbiota dysbiosis in mice.

### Binge-on-chronic alcohol consumption induced intestinal mycobiota dysbiosis

**Fig 3:**
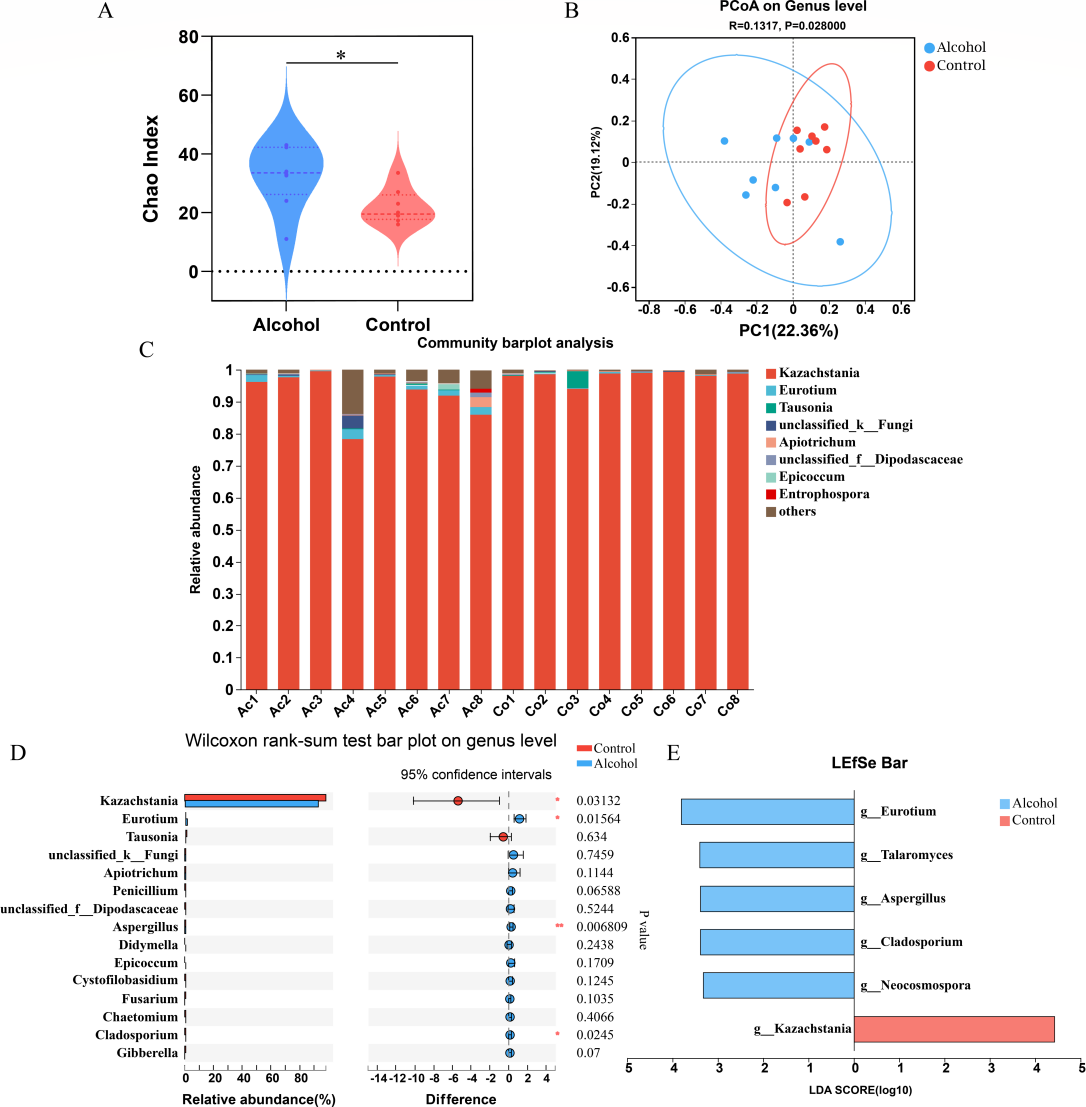
Binge-on-chronic alcohol consumption altered the intestinal fungal mycobiota in mice. (**A**) The α-diversity of the fungi at the genus level. (**B**)The PCoA diagram is based on the unweighted UniFrac distances. (**C**) The composition distribution of fungal genera in all samples. (**D**) The difference in abundance of the top 15 fungal genera between the two groups. (**E**) LDA > 3.0.

We also evaluated the effect of binge-on-chronic alcohol consumption on the intestinal fungal community by internal transcribed spacer (ITS) sequencing. The α-diversity showed that the Chao index significantly increased in the alcohol group when compared to the control group (Fig. 3A). PCoA analysis showed that the fungal community structures of the two groups were separated (*P* = 0.028, Fig. 3B). At the phylum level, *Ascomycota* and *Basidiomycota* were the dominant phylum in both groups (Table S3). At the genus level, *Kazakhstan* and *Eurotium* were the dominant genera in both groups (Fig. 3C). Changes in all fungal genera in the two groups are shown in Table S4. Compared with the control group, the relative abundances of *Eurotium*, *Aspergillus,* and *Cladosporium* significantly increased, while *Kazachstania* obviously decreased in alcohol groups (Fig. 3D). Moreover, LEfSe analysis also showed that the abundance of *Eurotium, Talaromyces,* and *Aspergillus* were relatively high-abundance species in alcohol group, while *Kazachstania* was relatively high-abundance species in control group (Fig. 3E). Figure S2 shows the cladogram of the different species between the two groups from phylum to genus level. All these results indicated that binge-on-chronic alcohol consumption induced gut fungi mycobiota dysbiosis.

### Binge-on-chronic alcohol consumption altered the trans-kingdom network construction of bacterial-fungal

**Fig 4:**
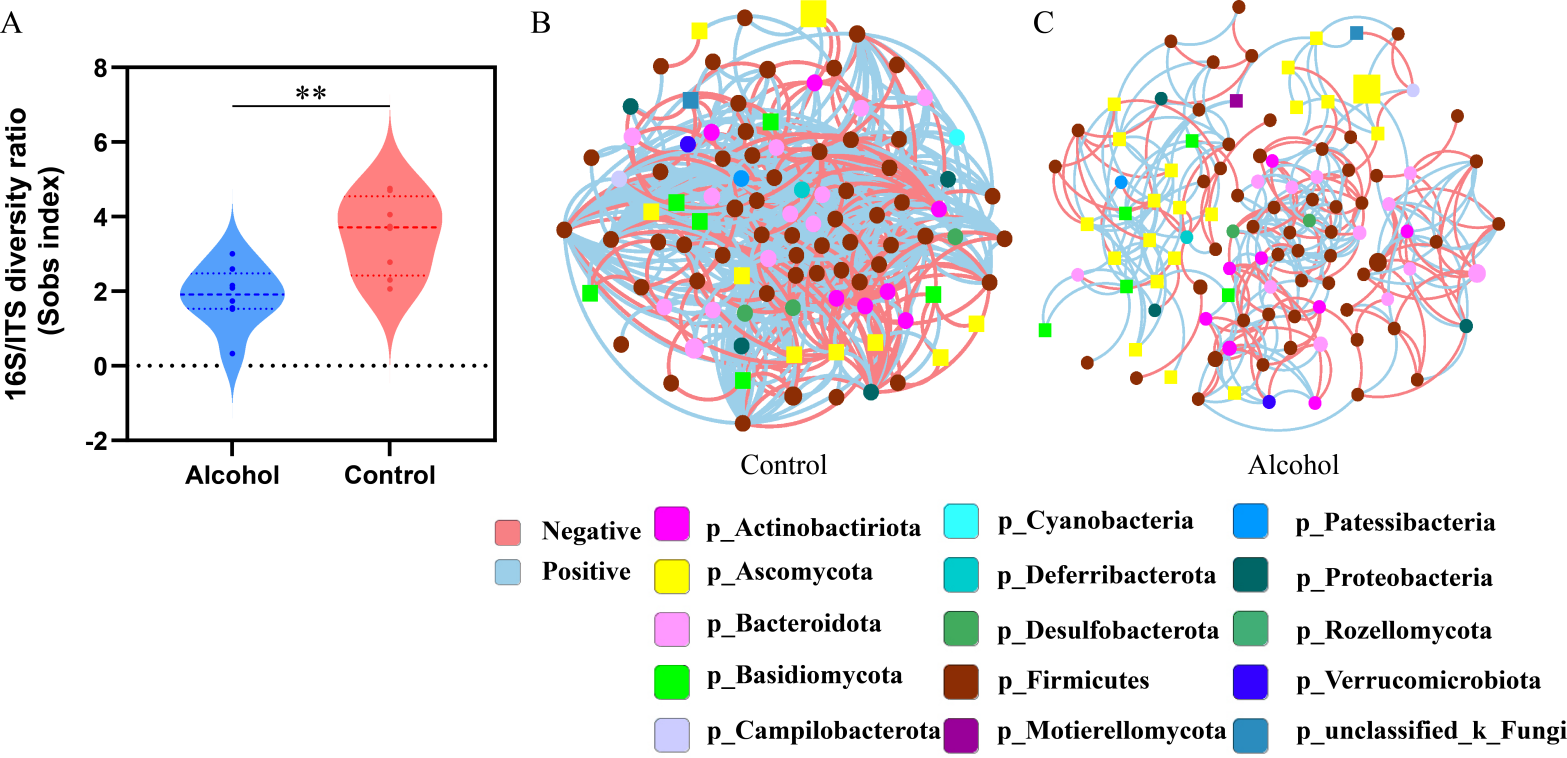
Binge-on-chronic alcohol consumption altered the trans-kingdom network construction of bacterial-fungal. (**A**) The diversity ratio of 16S/ITS. (**B**) The trans-kingdom abundance correlation networks in the control group at the genus level. (**C**) The trans-kingdom abundance correlation networks in the alcohol group at the genus level. Each node represents a genus, and the size of the node represents the average abundance of each genus. The squares represent fungi and the circles represent bacteria. Edges indicate different correlations, blue is a positive correlation and red is a negative correlation, and only the species with significant correlation (*P* < 0.05) were shown in the diagram.

The fungal-to-bacterial species ratio is one of the indicators of gut microbiome balance. To evaluate the effect of alcohol consumption on gut equilibrium, we analyzed the fungi-to-bacteria species ratio with 16S/ITS. The results showed that compared with the control group, the 16S/ITS ratio of the alcohol group significantly decreased (Fig. 4A), which indicated that chronic alcohol consumption disturbed the gut equilibrium between bacteria and fungi. Then, the trans-kingdom network analysis between bacteria and fungi was performed to assess the interplay at the genus level. The results showed that the trans-kingdom network was obviously different in the control group (Fig. 4B) vs the alcohol group (Fig. 4C). In the alcohol group, we found that the complexity of the network was notably reduced and the relationship between bacteria and fungi was also inconspicuous. However, in the control group, the bacteria and fungi were patently related to each other, gathering in a cluster and forming a more complex network (Fig. 4B). Compared with the control group, the number of fungal nodes in the alcohol group network increased from 16 to 28 (Table 1). However, this correlation is obviously weakened in the alcohol group. There were 488 negative correlation edges in the control group, but it reduced dramatically to 120 in the alcohol group (Table 1). The value of the coefficient of connectedness was obviously lower in the alcohol group (2.77) vs the control group (7.10). All these results suggested that alcohol consumption obviously altered the bacterial-fungal trans-kingdom network, reduced the interactions between bacteria and fungi, and resulted in gut microbiome dysbiosis.

**TABLE 1:** The parameters of the trans-kingdom networks of each group

Parameters	Control group	Alcohol group
Nodes	100	109
Fungi (F)	16	28
Bacteria (B)	84	81
Negative edges	488	120
F–F	5	1
F–B	43	22
B–B	440	97
Positive edges	222	182
F–F	3	36
F–B	40	25
B–B	179	111
Coefficient of connectedness	7.10	2.77

### Binge-on-chronic alcohol consumption altered the fecal metabolic profile

**Fig 5:**
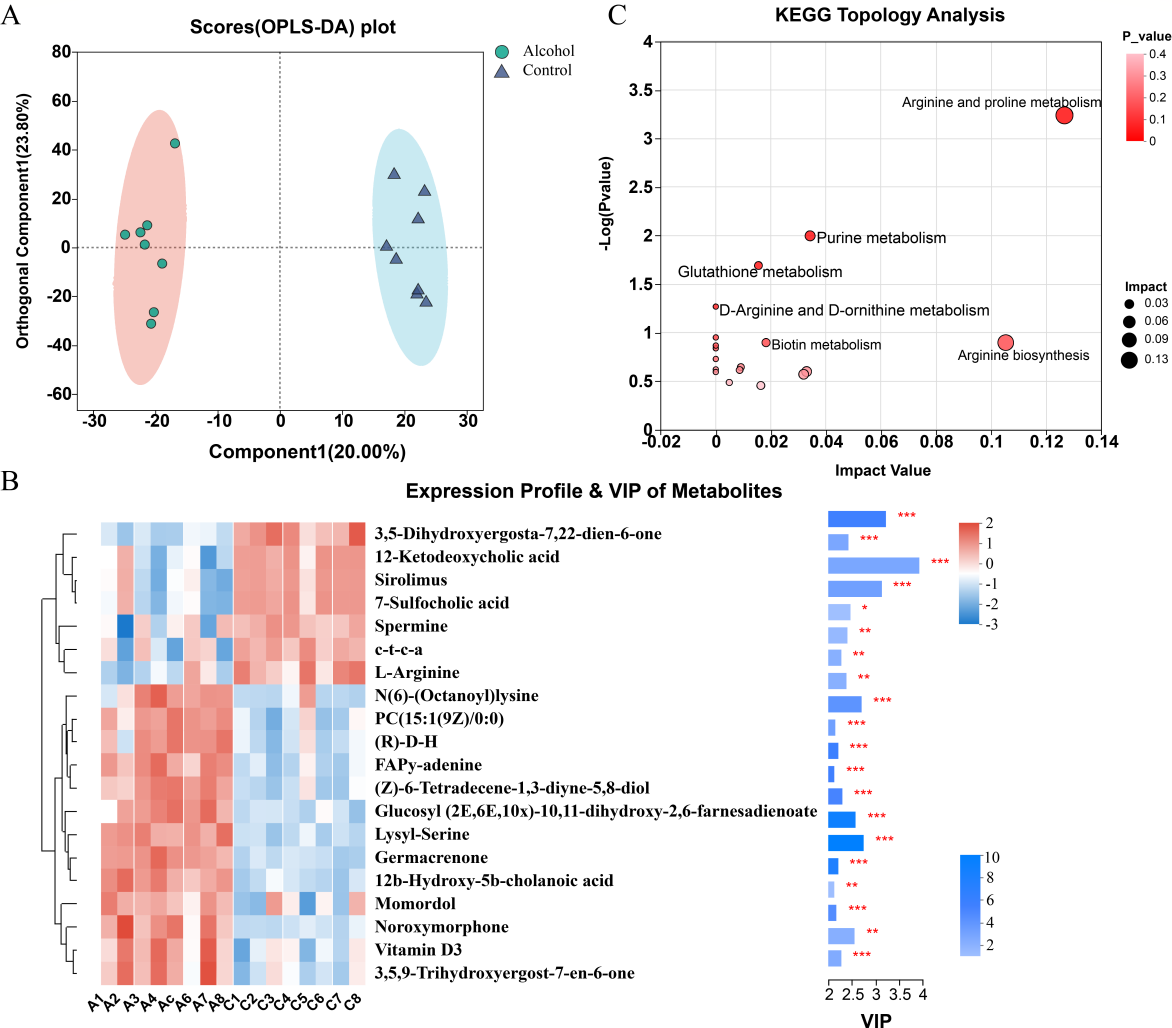
Untargeted metabolomics of the fecal metabolic profile. (**A**) OPLS-DA scores of fecal metabolites in the alcohol group and the control group. (**B**) Heat map of the top 20 significant differential metabolites between the alcohol and the control group. (**C**) The topology analysis of the KEGG metabolic pathway. *, *P* < 0.05; **, *P* < 0.01, ***, *P* < 0.001. c-t-c-a: 6-[2-carboxy-2-(hydroxymethyl)−2-methylethoxy]−3,4,5-trihydroxyoxane-2-carboxylic acid; (**R**)-D-H: (**R**)−3,4-dihydro-2-methyl-2-(4,8,12-trimethyl-3,7,11-tridecatrienyl)−2H-1-benzopyran-6-ol.

We used liquid chromatography and mass spectrometry (LC–MS) to assess the fecal metabolic profiles of the alcohol group and the control group mice. The orthogonal partial least squares discriminant analysis (OPLS-DA) (Fig. 5A) with its displacement test (Fig. S3) showed divergent trends of the fecal metabolic profiles between the alcohol group and the control group, suggesting that binge-on-chronic alcohol consumption had an obvious effect on the fecal metabolome. Based on the Kyoto Encyclopedia of Genes and Genomes (KEGG) database, we identified 169 metabolites with different abundances between the alcohol group and the control group. 90 differential metabolites in positive ion mode and 79 metabolites in negative ion mode were identified by parameters of *P* < 0.05, variable important in projection (VIP) > 1, and fold change > 1 (Fig. S4). According to a variable value with a VIP > 1 and *P* < 0.05 in the Wilcoxon rank-sum test, 20 fecal metabolic biomarkers were significantly altered between the two groups. The specific parameters of these significantly changed metabolites are shown in Table S5. Compared with the control group, we found that the expression level of 13 metabolites was up-regulated whereas that of 7 metabolites was down-regulated in the alcohol group (Fig. 5B). Interestingly, among these different metabolites, 12-ketodeoxycholic acid, and 7-sulfocholic acid belong to the secondary bile acids, which were significantly down-regulated in the alcohol group in comparison with the control group. Then, MetaboAnalyst was used to analyze the pathways involved in these metabolites. There are 18 metabolic pathways that were related to the metabolites in the alcohol and control group. According to the *P*-value and impact value, the pathways related most closely to alcohol consumption include arginine and proline metabolism (impact value = 0.13; *P* < 0.001), purine metabolism (impact value = 0.03; *P* < 0.05), and glutathione metabolism (impact value = 0.02; *P* = 0.020), which belong to amino acid metabolism pathways, nucleotide metabolism pathways, and metabolism of other amino acids pathways (Fig. 5C; Table S6). Taken together, these data revealed that chronic alcohol consumption significantly disturbed the feces metabolic profile and metabolic pathways in mice.

### Correlation analysis between the gut microbiome and feces metabolites

**Fig 6:**
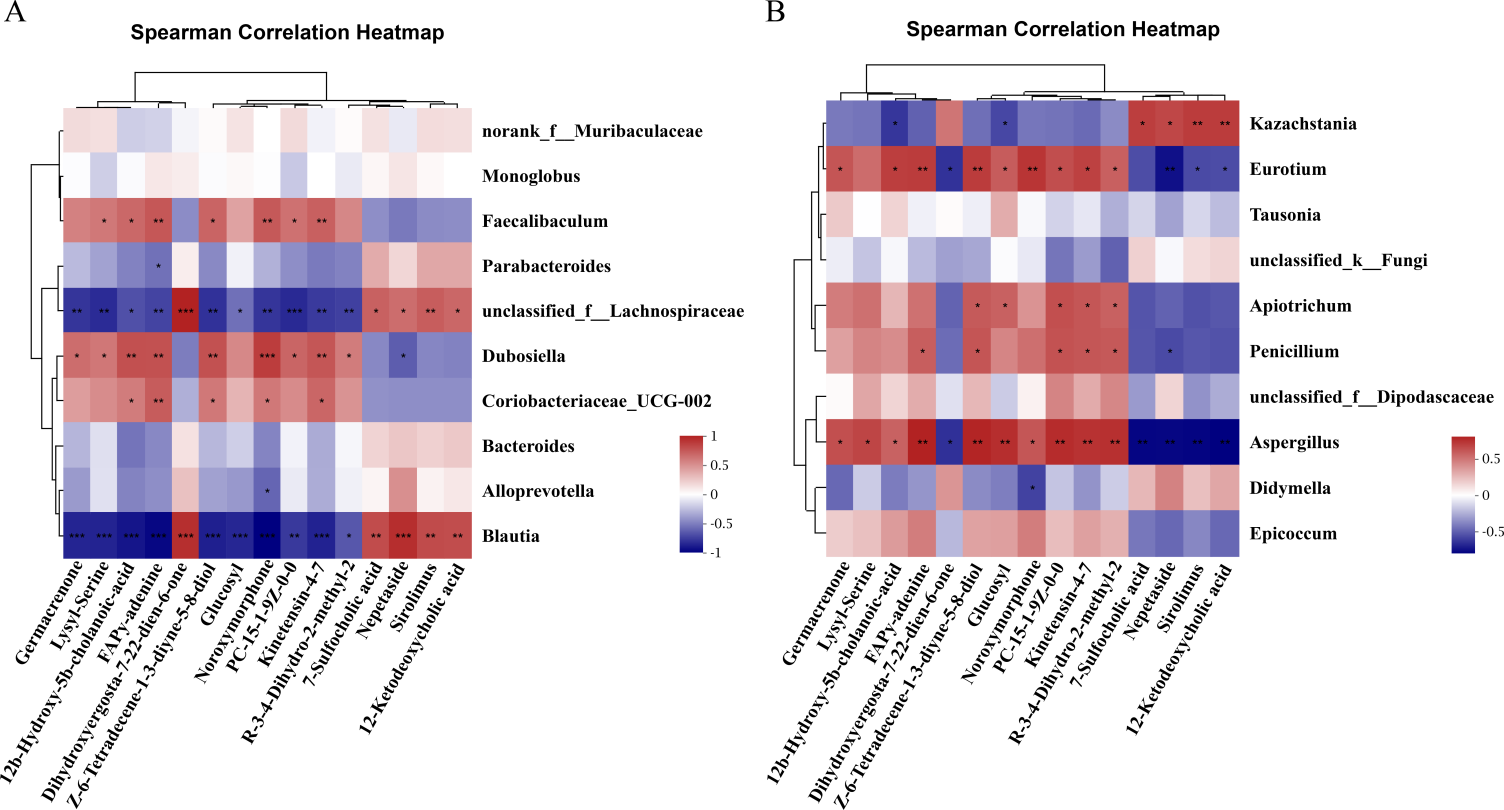
The heat map of Spearman’s rank correlation analysis. (**A**) The correlation between bacteria and metabolites. (**B**) The correlation between fungi and metabolites. *, *P* < 0.05, **, *P* < 0.01, ***, *P* < 0.001. Glucosyl: glucosyl (2E,6E,10x)−10,11-dihydroxy-2,6-farnesadienoate.

To investigate the potential correlations between the differential microbiota and metabolites, we performed two correlation analyses based on the Spearman correlation coefficients. We selected the 10 most abundant genera with 15 significantly different metabolites for correlation assessment by linear regression analysis. Notably, three of the 15 differential metabolites were secondary bile acids. Overall, in the correlation analysis between bacteria and metabolites (Fig. 6A), we identified 31 significant positive and 23 significant negative correlations. In particular, *Lachnospiraceae* and *Blautia* were strongly correlated with the secondary bile acids. *Unclassified_f_Lachnospiraceae* was positively correlated with 7-sulfocholic acid (R = 0.62, *P* = 0.01) and 12-ketodeoxycholic acid (R = 0.59, *P* = 0.02), respectively, and was negatively associated with 12b-hydroxy-5b-cholanoic acid (R = −0.61, *P* = 0.01). *Blautia* was positively correlated with 7-sulfocholic acid (R = 0.70, *P* < 0.01) and 12-ketodeoxycholic acid (R = 0.70, *P* < 0.01), respectively, and was negatively associated with 12b-hydroxy-5b-cholanoic acid (R = −0.82, *P* < 0.001). Figure 6B shows the results of correlation analysis between fungus and metabolites. In total, there were 33 significant positive and 13 significant negative correlations. We found that *Kazachstania* was positively correlated with 7-sulfocholic acid (R = 0.62, *P* = 0.01) and 12-ketodeoxycholic acid (R = 0.62, *P* = 0.01), and negatively correlated with 12b-hydroxyl-5b-cholanoic acid (R = −0.56, *P* = 0.02). Also, *Aspergillus* was negatively correlated with 7-sulfocholic acid (R = −0.70, *P* < 0.01) and 12-ketodeoxycholic acid (R = −0.71, *P* < 0.01), and positively correlated with 12b-hydroxyl-5b-cholanoic acid (R = 0.50, *P* = 0.05). In conclusion, the strong correlation between changes in gut microbiota and metabolites suggested that binge-on-chronic alcohol consumption induced gut microbiome dysbiosis, which then led to changes in feces metabolites.

**Fig 7:**
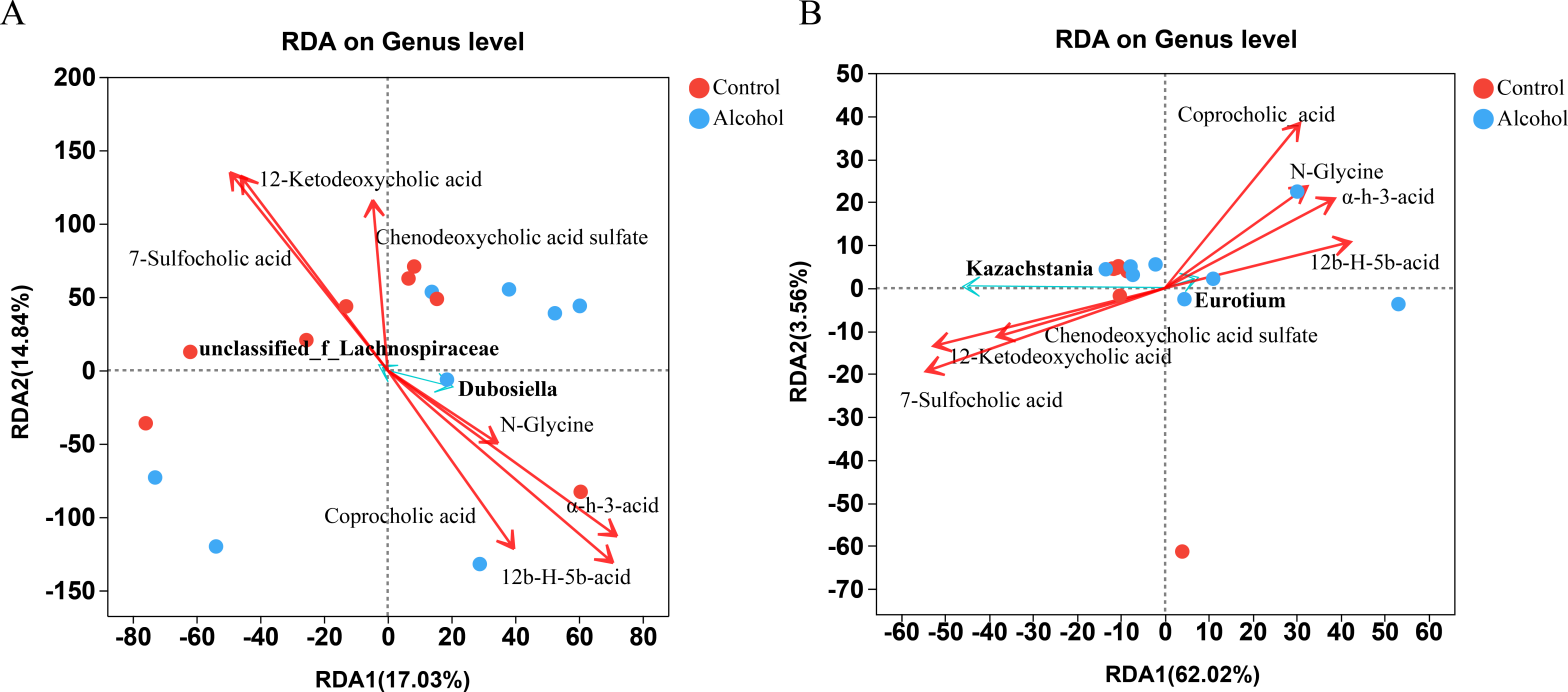
RDA association analysis of secondary bile acids and differential (**A**) bacteria and (**B**) fungi. The green and red dots in the figure represent samples from the alcohol and the control groups, respectively. The arrows from the origin indicate different environmental factors, and the length of the arrow indicates the degree of influence of that environmental factor on the species. The angles between the environmental factors represent the correlation between them, with acute angles indicating a positive correlation, obtuse angles indicating a negative correlation, and right angles indicating no correlation. α-h-3-acid: alpha-hydroxy-3oxochol-4en-24oic acid; N-glycine: N-[(3 a,5b,7a)−3-hydroxy-24-oxo-7-(sulfooxy)cholan-24-yl]-glycine; 12b-H-5b-acid: 12b-hydroxy-5b-cholanoic acid.

Since secondary bile acids are strongly associated with alterations in the intestinal microbiome, redundancy analysis (RDA) was used to further analyze the correlation between specific species and secondary bile acids identified in this study. As shown in Fig. 7A, 12b-hydroxyl-5b-cholanoic acid, and alpha-hydroxy-3oxochol-4en-24oic acid had a significant negative correlation with *Lachnospiraceae* and a positive correlation with *Dubosiella*. On the contrary, 7-sulfocholic acid had a positive correlation with *Lachnospiraceae* and a negative correlation with *Dubosiella*. The results of fungal RDA (Fig. 7B) showed that the 7-sulfocholic acid and 12-ketodeoxycholic acid were positively correlated with *Kazachstania* and negatively correlated with *Eurotium*. Coprocholic acid was negatively correlated with *Kazachstania* and positively correlated with *Eurotium*. Taken together, the above results indicated that the gut microbiome alteration induced by binge-on-chronic alcohol consumption was closely related to the changes in fecal metabolites, especially the changes in secondary bile acids and their derivatives.

### Secondary bile acids inhibited *K. pneumoniae* growth

**Fig 8:**
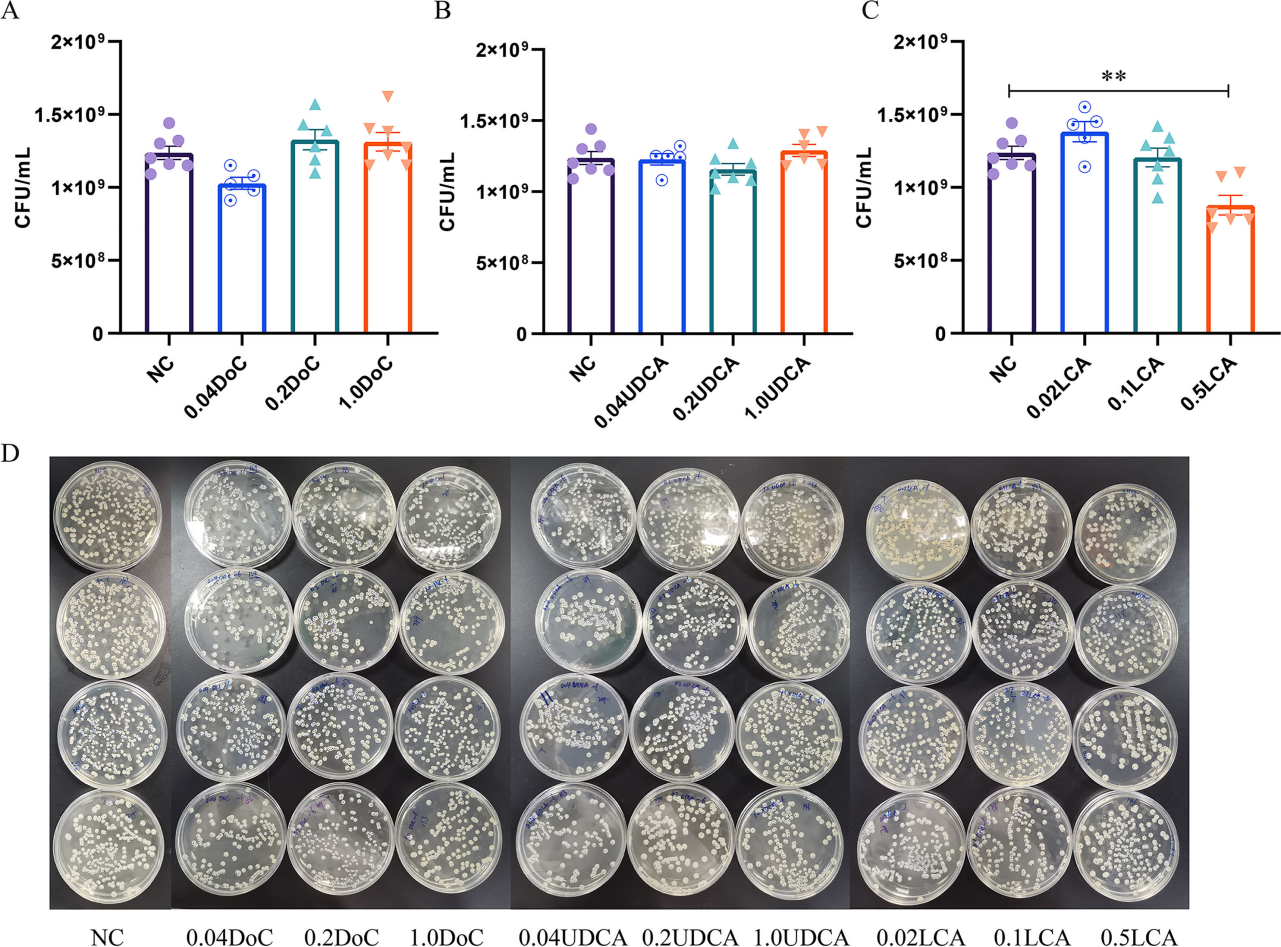
Secondary bile acid inhibited the growth of *K. pneumoniae*. The actual survival number of *K. pneumoniae* on Luria-Bertani (LB) agar plate after growing in a medium without secondary bile acid or with different concentrations of (**A**) DoC, (**B**) UDCA, and (**C**) LCA medium for 8 h. (**D**) Growth of *K. pneumoniae* on LB agar plates with different secondary bile acid treatments. Data are represented as means ± SEM. Statistical comparisons were made using one-way analysis of variance (ANOVA). *, *P* < 0.05, **, *P* < 0.01.

Based on the above results, we hypothesized that alcohol-induced dysbiosis of the gut microbiome, resulting in the alteration of bile acid metabolism, may contribute to increased *K. pneumoniae* colonization in the intestinal tract of mice. So, we performed a series of antibacterial tests *in vitro* to investigate the capacity of secondary bile acid against *K. pneumoniae*. The results showed that both deoxycholic acid (DoC) and ursodeoxycholic acid (UDCA) had no obvious effect on the growth of *K. pneumoniae* (Fig. 8A and B). However, lithocolic acid (LCA) could significantly inhibit the growth of *K. pneumoniae* with a concentration of 0.5 mg/mL (Fig. 8C).

### Lithocholic acid inhibited the adhesion of *K. pneumoniae* to Caco-2 cells

**Fig 9:**
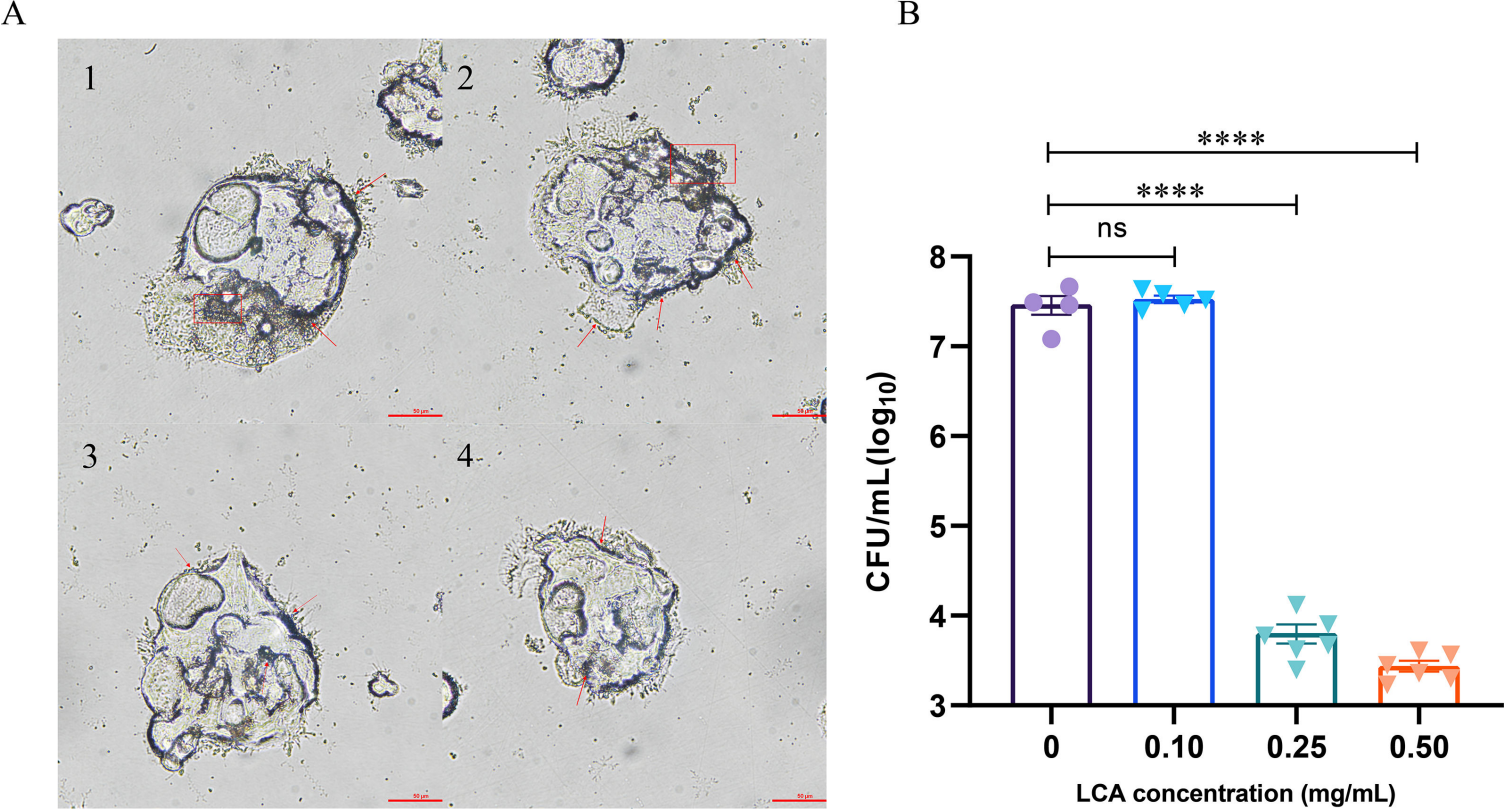
Effect of lithocholic acid on the adhesion of *K. pneumoniae* to Caco-2 cell. (**A**) *K. pneumoniae* adhering to Caco-2 cells (×400). “1”, the control group without LCA treatment, “2–4”, with the concentration of 0.1 mg/mL, 0.25 mg/mL, and 0.5 mg/mL LCA, respectively. (**B**) The histogram of LCA treated with different concentrations. Statistical analysis was performed using one-way ANOVA. *, *P* < 0.05, **, *P* < 0.01, ***, *P* < 0.001.

Based on the bacteriostatic ability of this secondary bile acid against *K. pneumoniae*, we chose LCA to investigate its effect on the adhesion of *K. pneumoniae* to Caco-2 cells. First, decreased adhesion of LCA-pretreated *K. pneumoniae* to Caco-2 cells was observed by microscope (Fig. 9A). Compared with the group without bile acid treatment, the adhesion abilities of *K. pneumoniae* to Caco-2 cells treated with LCA (0.25 and or 0.5 mg/mL) were decreased significantly. The results were further confirmed by CFU counting. As shown in Fig. 9B, compared with the control group, LCA (0.1 mg/mL) pretreatment had no obvious effect on the adhesion of *K. pneumoniae* to Caco-2 cells. However, a higher concentration of LCA (0.25 mg/mL or 0.5 mg/mL) could significantly inhibit *K. pneumoniae* adhesion to Caco-2 cells, and this inhibition ability is dose-dependent.

## DISCUSSION

A strong relationship between alcohol consumption and gut microbiota dysbiosis has been consistently found in animal and human models after alcohol consumption (28, 29). Binge-on-chronic alcohol consumption disrupts the balance of intestinal microbiota in mice, as shown in this study by an elevation of *Faecalibaculum*, *Dubosiella, Eubacterium*, and *Akkermansia*, as well as a decrease in *Blautia*, *Lachnospiraceae*, *Desulfovibrionaceae*, and *Streptococcus*. Similar to previous results, chronic alcohol consumption reduced *Akkermansia* (30–32) and *Roseburia* spp. (32, 33) but increased the genus *Enterococcus* (31, 34). Certain results in this study were contrary to published studies. For example, previous studies displayed an increased level of *Streptococcus* in patients with alcohol use disorders (30, 34), but in this study, *Streptococcus* was decreased in alcohol-fed mice. These may be related to the variety of experimental animals and the different intervention times.

We know that chronic alcohol consumption is related to intestinal bacterial disorder. However, the relationship between intestinal fungal and alcohol consumption is far from well understood. In alcohol-fed mice, it was observed that fungal diversity increased, featuring the decrease of *Kazachstania* and the increase of *Eurotium*, *Aspergillus*, and *Talaromyces*. Contrary to previous studies on alcoholic liver diseases, this study reveals that alcohol consumption leads to a decrease in fungal diversity (35). In addition, *Candida* overgrowth was found in most alcohol consumption studies (35–38) but not in the present one. For the above differences in intestinal microbiota, we speculate that the possible reasons for these differences might include the alcohol model (chronic, acute, or binge-on-chronic alcohol consumption), experimental animals (mice or rats), alcohol concentration (alcohol liquid diet or alcohol gavage), and the time of alcohol model (days to months).

Chronic alcohol consumption affects the composition of intestinal microbiota and leads to the destruction of homeostasis, while intestinal dysbiosis inevitably affects intestinal metabolome. Studies have shown that short-chain fatty acids, amino acids, and bile acids have all been altered significantly after alcohol consumption (39, 40). In addition, some metabolites such as fatty acids, steroids, lipids, and carnitine have also demonstrated changes to some extent (41, 42). Being consistent with published studies, this study also found alterations in amino acids, steroids, and lipids. Particularly, it demonstrated a dramatic change in secondary bile acid. Alcohol consumption significantly decreased secondary bile acid 7-sulfocholic acid and 12-ketodeoxycholic acid, while 12b-hydroxy-5b-cholanoic acid increased significantly. Primary bile acid combines with taurine or glycine to form bile salt secreted into the intestinal lumen, which was further transformed into secondary bile acid by intestinal microbiota. Secondary bile acid is closely related to intestinal microbiota, so we further explored the effect of secondary bile acid on intestinal colonization of *K. pneumoniae*.

Previous studies have found that secondary bile acids can affect the colonization of pathogenic bacteria. Thanissery et al. recently found that many secondary bile acids were able to inhibit TCA-mediated *Clostridium difficile* spore germination and outgrowth (43). Another study by Vidal demonstrated that at a physiological concentration, DoC killed all tested *S. pneumoniae* strains 2 h post-inoculation (44). In addition, Guinan found that LCA and DCA, at *in vivo* cecal micelle concentrations, inhibit *Candida albicans* growth, germ tube, hyphae, and biofilm formation *in vitro* (45). Based on previous studies, we hypothesized that secondary bile acids may have direct antibacterial activity against *K. pneumoniae* and may play a key role in maintaining the colonization resistance of the gastrointestinal tract to *K. pneumoniae*. To clarify whether secondary bile acids have antibacterial activity against *K. pneumoniae*, we conducted *in vitro* studies to determine whether physiological concentration and the higher than the physiological concentration of secondary bile acids can inhibit the growth and cell adhesion of *K. pneumoniae*.

In our study, we proved that LCA, as a secondary bile acid, could effectively inhibit the growth of *K. pneumoniae* above physiological concentration. Since we found that LCA can effectively inhibit the growth of *K. pneumoniae*, we further studied whether LCA affects the adhesion of *K. pneumoniae* to intestinal epithelial cells. As a result, we discovered that LCA at high concentration could significantly inhibit the adhesion of *K. pneumoniae* to intestinal epithelial cells Caco-2, and the inhibitory effect of LCA was more conspicuous with the increase in concentration. The above results *in vitro* suggest that LCA can effectively inhibit the growth of *K. pneumoniae* and prevent its colonization in the intestinal tract. The latest study found that nitrofuran-deoxycholate synergy against Gram-negative bacteria, including *K. pneumoniae* (46). Therefore, some secondary bile acids (LCA) may be used as a potential drug to prevent the colonization and spread of *K. pneumoniae*.

### Study limitations

This study has some limitations that need to be mentioned. First, due to the expense of multi-omics analysis, a relatively small sample of mice was used in this study, which might have caused the neglect of some important differences in the gut bacterial and fungi microbiota and fecal metabolite profiles between groups. Second, this study lacks further experiments to prove the direct causal relationship between alcohol-induced gut microbiota dysbiosis and increased *K. pneumoniae* colonization. Hence, the use of fecal microbiota transplantation methods or the inoculation of specific bacteria and fungi to verify this point is a future research topic. Third, it is not clear whether supplementing some secondary bile acids could inhibit *K. pneumoniae* colonization *in vivo*. Thus, future studies need to establish a *K. pneumoniae* infection animal model with secondary bile acid intervention to resolve this limitation of the study.

### Conclusions

In summary, our data reveal that chronic alcohol consumption could induce intestinal microbiota dysbiosis, which is demonstrated through not only gut bacteria but also gut fungi. Gut microbiota dysbiosis results in the disorder of metabolome, especially the metabolism of bile acid metabolic balance. The level of some secondary bile acids (including LCA) decreased. Higher concentrations of LCA can protect against intestinal colonization of *K. pneumoniae* by inhabiting the bacterial growth and adhesion to the host cells. Hence, regulating the balance of gut microbiota and intestinal bile acid metabolism may be a potential strategy for preventing and treating *K. pneumoniae* infection.

## MATERIALS AND METHODS

### Animals and ethics statement

Twenty-four male SPF C57BL/6 mice (8 to 10 weeks old) were purchased from SKBEX Biotechnology Co., Ltd. (Anyang, China; No. LISC20200005). Mice were maintained in a standard room with a temperature of 20–22°C and a relative humidity of 50% ± 5%. Animals were housed in filter-topped cages, six mice in each cage, and were provided food and autoclaved water *ad libitum*. All experiments were conducted according to the Declaration of Helsinki and were approved by the Animal Care and Use Ethics Committee of Xinxiang Medical University (No. XYLL-20210319).

### The binge-on-chronic alcohol model of mice

The binge-on-chronic alcohol model was established according to the National Institute for Alcohol Abuse and Alcoholism (NIAAA) chronic-binge alcohol model with some modifications (47). Briefly, after acclimated to a liquid diet for 6 days using a control liquid diet without alcohol (Cat#: TP4010C, TROPHIC Animal Feed High-tech Co, Ltd., Nantong, China), mice were randomly divided into two groups (8–9 mice in each group), including a control group and an alcohol group. During the experimental procedure, the control group was fed a control liquid diet without alcohol (Cat#: TP4010C), whereas the alcohol group received a 5% ethanol liquid diet (Cat#: TP4010A). The volume of the control liquid diet in the control group was adjusted daily based on the consumption of a 5% ethanol liquid diet in the alcohol group. Mice were maintained on the 5% ethanol liquid diet (or control liquid diet) for 15 days to establish the binge-on-chronic alcohol model. On the 7th and 14th day of the experiment, mice in the alcohol group were administrated 4 g/kg alcohol (24.03%, vol/vol) by gavage (binge). To ensure the same caloric intake, mice in the control group were given 9 g/kg maltodextrin (45%, wt/vol) by gavage.

### *K. pneumoniae* infection and quantification

*K. pneumonia*e (strain Kp-13, ST23), isolated and preserved by our laboratory, were grown in LB broth (Beijing Aoboxing Universeen bio-tech co. Ltd., Beijing, China) in a shaking incubator (37°C, 200 rpm) for 16 h. *K. pneumoniae* were collected by centrifugation with 8,000 rpm for 10 min at 4°C, washed with PBS, then resuspended in PBS at the concentration of 1 × 10^9^ CFU/mL. Twenty-four hours after the final alcohol binge, all mice were infected with *K. pneumoniae* (2 × 10^8^ CFU, 200 µL each mouse) by gavage. To quantify bacterial loads in the gastrointestinal tract, animal feces were collected at 24 h and 48 h after infection with *K. pneumoniae*. These feces were weighed and homogenized with PBS, and the actual number of bacteria was continuously gradient diluted to *Klebsiella* selective agar plates (MacConkey inositol adonitol carbenicillin agar, Qingdao Haibo Biology Technology Co., Ltd., Qingdao, China) for standard colony count.

### Fecal DNA extraction, 16S rRNA, and ITS sequencing

Currently, stool samples still have been the main source of information for gut microbiome studies. So stool samples were collected and total genomic DNA of the gut microbiome was extracted from different feces samples using the E.Z.N.A. soil DNA Kit (Omega Bio-tek Biotechnology Co., Ltd., Norcross, GA, USA) according to the manufacturer’s instructions. The integrity and concentration of DNA were determined by 1.0% agarose gel electrophoresis and a NanoDrop ND-2000 spectrophotometer (Thermo Scientific Inc., USA), respectively. The V3–V4 region of bacterial 16S rRNA gene was amplified using primers 338F (5′-ACTCCTACGGAGGCAGCAGCAGCA-3′) and 806R (5′-GGACTACHVG GTWTAAT-3′). The ITS region of fungi was amplified using primers ITS1F (5′-CTTGGTCATT TAGAGGAAGTAA-3′) and ITS2R (5′-GCTGCGTTCTTCATCGATGC-3′). These amplification reactions were completed by ABI GeneAmp 9700 PCR thermocycler (ABI, CA, USA). The PCR product was extracted and purified, and then the amplicons were pooled in equimolar amounts and paired-end sequenced on an Illumina MiSeq PE300 platform (Illumina, San Diego, USA) according to the standard protocols by Majorbio Bio-Pharm Technology Co. Ltd. (Shanghai, China).

### Untargeted metabolomics analysis of fecal metabolome

The fecal supernatant samples were dissolved, ground, ultrasonicated, incubated, and centrifuged according to the previous document (48, 49). A quality control assessment of stability during the experiment was conducted by combining 10 µL of supernatant from each sample. Then, the metabolic profiles of all fecal samples were investigated using LC–MS. Detailed chromatographic and MS conditions have been described previously (50). The MS raw data were extracted by ProteoWizard (v3.0.9134) and the XCMS package in R (v3.2) to obtain the characteristic peak information contained the retention time, the mass-to-charge ratio value, and peak intensity. The databases of the Kyoto Encyclopedia of Genes and Genomes (KEGG; http://www.genome.jp/kegg) were used to identify the metabolites.

### Inhibition of secondary bile acids against *K. pneumoniae*

According to the reports of Hamilton and Thanissery et al. (43, 51), secondary bile acids with three concentrations were used to carry out bile acid challenge experiments. Briefly, 5 mL of LB broth with or without bile acids of different concentrations was inoculated with *K. pneumoniae* cultured overnight. After incubation (37°C, 200 rpm, 8 h), standard colony count was used to determine the number of viable bacteria by plating serial dilutions onto LB agar. The secondary bile acids including deoxycholic acid (Cas#: No.302-95-4), ursodeoxycholic acid (Cas#: No. 128-13-2), and lithocholic acid (Cas#: No. 434-13-9) were purchased from McLean Biochemical Technology Co., Ltd.

### Adhesion assay of *K. pneumoniae* to Caco-2 cell

Caco-2 cells (ATCC number: HTB-37) were inoculated into 24-well plates or 12-well plates in minimum essential medium (MEM) with 20% FBS at a seeding density of 2 × 10^5^ cells/well (24-well plate) or 5 × 10^5^ cells/well (12-well plate). One hour before the adhesion experiments, the medium was replaced with prewarmed MEM without penicillin and streptomycin. *K. pneumoniae* at exponential growth stage were centrifuged and washed three times with prewarmed MEM. The bacteria were diluted with MEM (containing three secondary bile acids) and added to the Caco-2 monolayers to make the multiplicity of infection (MOI) 100:1. After Caco-2 cells incubated with *K. pneumoniae* for 4 h, the wells were washed three times with PBS to remove unattached bacteria. Caco-2 cells were then lysed with 1% TritonX-100 (Shanghai Beyotime Biotechnology Co., Ltd., Shanghai, China) to prepare appropriate dilutions, which were plated on LB agar plates incubated (16 h, 37°C) and counted.

To better observe the adhesion ability of *K. pneumoniae* to Caco-2 cells, we carried out the following experiment. A volume of 2 mL of *K. pneumoniae* pre-treated with indicated bile acids was added to the 12-well plate containing 5 × 10^5^ cells/well to make the MOI 100 :1. After incubating for 4 h, MEM containing *K. pneumoniae* was blown, and then each cell culture hole was washed three times with PBS, fixed with 4% paraformaldehyde (Beijing Solarbio Technology Co., Ltd., Beijing, China) for 30 min, dried naturally and observed under microscope (400×) (Nikon Corporation, Japan).

### Bioinformatics statistical analysis

All the bioinformatics data were analyzed on the free online platform of Majorbio Cloud Platform (https://www.majorbio.com/). The raw reads of 16srRNA and ITS sequencing were filtered by DADA2 in Quantitative Insights Into Microbial Ecology (QIIME2 v.2018) quality filters. The amplicon sequence variants (ASVs) were classified according to the relative database (silva138/16 s_bacteria; unite8.0/its_fungi) at 99% sequence similarity, at 70% confidence using the Ribosomal Database Project Classifier 2.8. The alpha diversity was assessed according to the inverse Simpson and Chao indices for microbiome bioinformatics analysis. The beta-diversity indices (analyzed using PCoA) were calculated based on unweighted UniFrac distances at the ASV level. A permutational analysis of variance was performed to assess the variation in the taxonomic structure of microbiota communities between groups. Linear discriminant analysis (LDA) and LEfSe analyses were performed to compare biomarkers between groups. For metabolites analysis, after normalizing the raw data, principal component analysis and orthogonal to partial least-squares discriminate analysis were employed to characterize metabolic perturbation and differences among groups. The different metabolites’ pathway topology was analyzed with MetaboAnalyst version 3.0. Correlation analyses were performed using Spearman’s rho correlation test. The correlations among main gut bacterial genera, fungi genera, and fecal metabolites were assessed by linear regression analysis adjusted for alcohol consumption. The trans-kingdom network figures were built using the package igraph (version 1.2.6). All statistics were performed using SPSS version 22.0, and graphs were made with GraphPad Prism 9 or R package (version 3.6.2). If the data followed a normal distribution, unpaired Student’s *t*-tests were used to compare various parameters between the two groups. If the data did not follow a normal distribution, a non-parametric Wilcoxon rank sum test was used to compare the results. *P*-values of <0.05 were set as a threshold for statistical significance.

## Data Availability

The data sets generated for this study can be found in the Sequence Read Archive (SRA) of the National Center for Biotechnology Information (NCBI), BioProject number PRJNA1006278.

## SUPPLEMENTAL MATERIAL

Supplemental tables and figures msystems.00052-24-s0001.pdf Tables S1-S6 and Figures S1-S4.

Abstract msystems.00052-24-s0002.pdf Graphical abstract.

ASM does not own the copyrights to Supplemental Material that may be linked to, or accessed through, an article. The authors have granted ASM a non-exclusive, world-wide license to publish the Supplemental Material files. Please contact the corresponding author directly for reuse.
