# Supplementary material for: Alcohol-induced gut microbiome dysbiosis enhances the colonization of Klebsiella pneumoniae on the mouse intestinal tract
Source: mSystems. 2024 Feb 12;9(3):e00052-24. doi: 10.1128/msystems.00052-24 (PMC10949497; doi:10.1128/msystems.00052-24)
Supplement: Supplemental tables and figures — Tables S1-S6 and Figures S1-S4. [file msystems.00052-24-s0001.pdf]

Supplementary information

Figure. S1 LEfSe analysis from phylum to genus level of intestinal microbiota between the alcohol and control groups.

Figure. S2 LEfSe analysis from phylum to genus level of intestinal fungi between alcohol and control groups.

Figure. S3 The Orthogonal Projections to Latent Structures-Discriminant Analysis (OPLS-DA) model permutation test of the different metabolites.

Figure. S4 The volcano plot of the different metabolites between alcohol and control groups.

Table. S1 The relative abundance of bacterial microbiota in alcohol and control groups at the phylum level.

Table. S2 The relative abundance of bacterial microbiota in alcohol and control groups at the genus level.

Table. S3 The relative abundance of fungal mycobiota in alcohol and control groups at the phylum level.

Table. S4 The relative abundance of fungal mycobiota in alcohol and control groups at the genus level.

Table. S5 The differentially expressed metabolites between alcohol and the control group.

Table. S6 The metabolism pathway enrichment analysis.

Figure. S1 LEfSe analysis from phylum to genus level of intestinal microbiota between the alcohol and control groups.

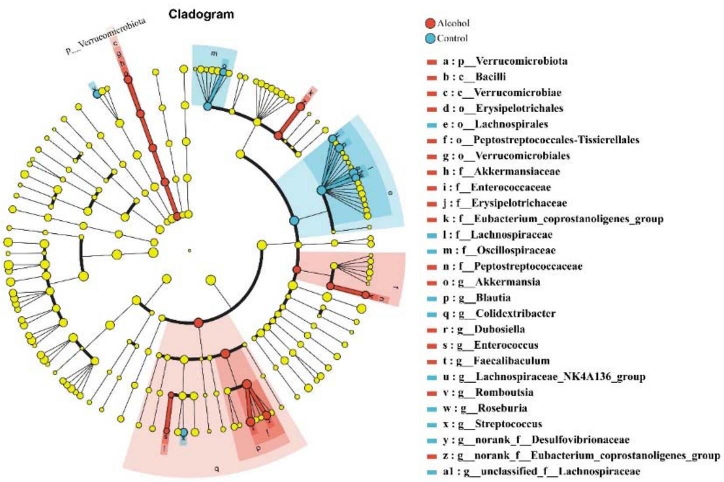

Figure. S2 LEfSe analysis from phylum to genus level of intestinal fungi between alcohol and control groups.

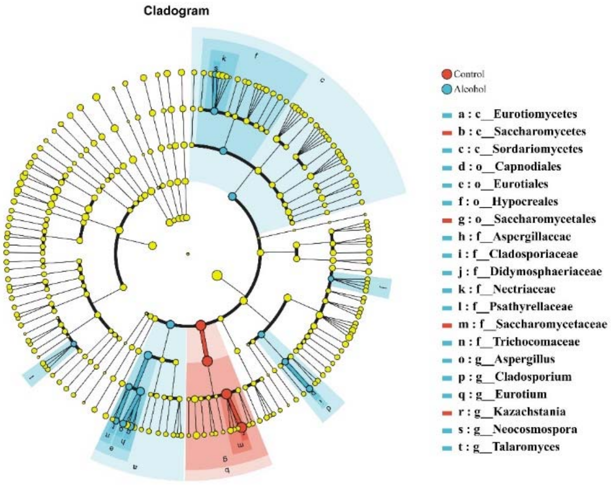

Figure. S3 The Orthogonal Projections to Latent Structures-Discriminant Analysis (OPLS-DA) model permutation test of the different metabolites. (A)positive ion and (B) negative ion modes

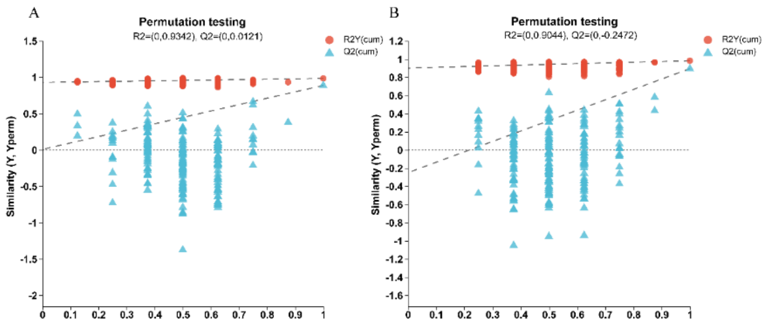

Figure. S4 The volcano plot of the different metabolites between alcohol and control groups. (A)positive ion and (B) negative ion modes

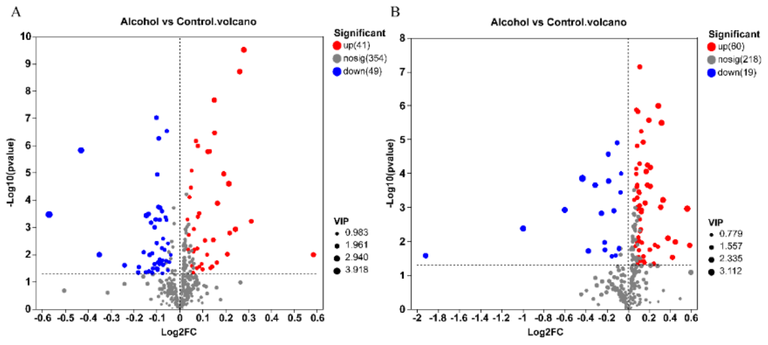

Table. S1 The relative abundance of bacterial microbiota in alcohol and control groups at the phylum level.

Species name	Alcohol-mean (%)	Control-mean (%)	P value
p__Firmicutes	48.53	46.67	0.713
p__Bacteroidota	36.36	42.75	0.495
p__Actinobacteriota	8.359	4.505	0.18
p__Proteobacteria	2.869	1.146	0.083
p__Desulfobacterota	1.337	2.597	0.104
p__Campilobacterota	0.5954	1.417	0.636
p__Verrucomicrobiota	1.71	0.1085	0.002
p__Deferribacterota	0.2191	0.6138	0.066
p__Patescibacteria	0.02099	0.09932	0.534
p__Cyanobacteria	0	0.1029	0.032

Table. S2 The relative abundance of bacterial microbiota in alcohol and control groups at the genus level.

Species name	Alcohol-mean (%)	Control-mean (%)	P value
g__norank_f__Muribaculaceae	20.61	20.47	1
g__Monoglobus •	17.6	14.68	0.8748
g__Faecalibaculum	10.63	5.717	0.05203
g__Parabacteroides	6.443	9.581	0.2271
g__unclassified_f__Lachnospiraceae	3.989	6.273	0.007406
g__Dubosiella	7.179	2.494	0.01813
g__Coriobacteriaceae_UCG-002	6.533	2.479	0.1278
g__Bacteroides	3.704	5.235	0.3184
g__Alloprevotella	1.785	3.797	0.2701
g__Blautia	0.4213	4.048	0.0009391
g__Parasutterella	2.808	1.097	0.09241
g__norank_f__Desulfovibrionaceae	0.6031	2.399	0.03132
g__norank_f__Eubacterium_coprostanoligenes_group	2.584	0.341	0.005351
g__Lachnospiraceae_NK4A136_group	0.2652	2.082	0.000939
g__Alistipes	1.202	0.9123	0.4948
g__Lactobacillus	0.5334	1.547	0.8334
g__Helicobacter	0.5954	1.417	0.6363
g__Enterorhabdus	0.7126	1.209	0.7525
g__Akkermansia	1.71	0.1085	0.001874
g__Roseburia	0.258	1.521	0.03132
g__Rikenellaceae_RC9_gut_group	0.6599	1.023	0.2271

g__Streptococcus	0.04556	1.536	0.005385
g__norank_f__norank_o__Clostridia_UCG-014	1.432	0.01587	0.08926
g__Butyricimonas	0.7474	0.6696	0.6365
g__unclassified_f__Atopobiaceae	0.7331	0.5442	1
g__Muribaculum	0.6665	0.3706	0.9161
g__Desulfovibrio	0.7336	0.1869	0.01359
g__Colidextribacter	0.1582	0.7259	0.01796
g__Romboutsia	0.8744	0.003584	0.000778
g__unclassified_f__Oscillospiraceae	0.2324	0.644	0.1149
g__Mucispirillum	0.2191	0.6138	0.06608
g__Anaerotruncus	0.129	0.6973	0.06588
g__norank_f__Oscillospiraceae	0.1183	0.5473	0.05203
g__Christensenellaceae_R-7_group	0.258	0.2452	1
g__Odoribacter	0.277	0.192	0.6742
g__Rikenella	0.1561	0.3067	0.1149
g__norank_f__Ruminococcaceae	0.1531	0.2473	0.1547
g__Harryflintia	0.06041	0.3389	0.01345
g__Lachnoclostridium	0.07014	0.2596	0.001933
g__Oscillibacter	0.03788	0.2892	0.01352
g__Tuzzerella	0.0686	0.2473	0.1015
g__Lactococcus	0.2933	0	0.00453
g__ASF356	0.02969	0.2621	0.02354
g__Erysipelatoclostridium	0.03635	0.236	0.01306
g__Eubacterium_nodatum_group	0.2073	0.06143	0.3713
g__Parvibacter	0.1234	0.1152	0.8747
g__unclassified_f__Prevotellaceae	0.05017	0.1863	0.04552
g__norank_f__Peptococcaceae	0.05375	0.1618	0.05134
g__norank_f__norank_o__Clostridia_vadinBB60_group	0.04863	0.1418	0.09049
g__norank_f__Erysipelotrichaceae	0.09573	0.08857	0.8746
g__Adlercreutzia	0.06553	0.1183	0.3692
g__unclassified_f__Ruminococcaceae	0.01894	0.1613	0.06953
g__Gordonibacter	0.1577	0.006655	0.001759
g__Negativibacillus	0.0215	0.1428	0.2074
g__Veillonella	0.03942	0.1234	0.06678
g__norank_f__Lachnospiraceae	0.07986	0.05427	0.958
g__unclassified_c__Clostridia	0.06655	0.0645	0.6355
g__Candidatus_Saccharimonas	0.02099	0.09932	0.5338
g__Eubacterium_brachy_group	0.05938	0.06041	0.9577
g__Intestinimonas	0.009727	0.1049	0.008404
g__norank_f__norank_o__Gastranaerophilales	0	0.1029	0.03247
g__unclassified_f__Erysipelotrichaceae	0.03993	0.05222	0.9157
g__GCA-900066575	0.03123	0.04966	0.1853
g__norank_f__UCG-010	0.043	0.02662	0.5965

g__Marvinbryantia	0.008703	0.04761	0.05915
g__Mycoplasma	0.01331	0.04198	0.5009
g__unclassified_f__Rikenellaceae	0.05222	0	0.03247
g__norank_f__Eggerthellaceae	0.02457	0.02713	0.5895
g__Peptococcus	0.01638	0.03481	0.6944
g__Clostridium_sensu_stricto_1	0.006143	0.03379	0.03464
g__Candidatus_Soleaferrea	0.02867	0.01024	0.06953
g__UCG-009	0.01229	0.02457	0.241
g__Escherichia-Shigella	0.02611	0.009727	0.07769
g__norank_f__norank_o__Rhodospirillales	0.01024	0.02457	0.2516
g__Enterococcus	0.03276	0.002048	0.003511
g__Lachnospiraceae_FCS020_group	0.006655	0.02304	0.2845
g__norank_f__Christensenellaceae	0.01075	0.01792	0.1165
g__Paludicola	0.02048	0.007167	0.3086
g__A2	0.001024	0.02611	0.08474
g__Bacillus	0	0.02611	0.3816
g__NK4A214_group	0.01485	0.007679	0.9491
g__norank_f__norank_o__norank_c__Clostridia	0.01587	0.005119	0.4507
g__Butyricicoccus	0	0.01945	0.07645
g__Ralstonia	0.008703	0.01024	0.8222
g__Turicibacter	0.01587	0	0.07598
g__Ruminococcus_torques_group	0.01433	0	0.3816
g__norank_f__Flavobacteriaceae	0.005119	0.008191	0.1952
g__Lachnospiraceae_UCG-006	0.006143	0.007167	0.9117
g__Bilophila	0.0005119	0.01024	0.08441
g__unclassified_c__Bacilli	0	0.01024	0.3816
g__Acinetobacter	0.01024	0	0.3816
g__Anaerovorax	0.005119	0.004096	1
g__Family_XIII_AD3011_group	0.007167	0.001536	0.2697
g__Anaerofustis	0.001536	0.006655	0.1835
g__norank_f__Atopobiaceae	0.005631	0.001536	0.2697
g__UBA1819	0.00256	0.004096	1
g__Acetatifactor	0.002048	0.004096	0.5874
g__UCG-004	0.006143	0	0.3816
g__Bradyrhizobium	0.001536	0.003584	1
g__Bifidobacterium	0	0.004607	0.1709
g__unclassified_o__Bacteroidales	0.003584	0	0.1709
g__Lachnospiraceae_UCG-010	0	0.003584	0.1709
g__Staphylococcus	0	0.00256	0.3816
g__Prevotellaceae_UCG-001	0.00256	0	0.3816
g__Corynebacterium	0.00256	0	0.3816
g__Aquabacterium	0.002048	0	0.3816
g__Alkanindiges	0.002048	0	0.3816
g__unclassified_f__Anaerovoracaceae	0.001024	0	0.3816

g__norank_f__norank_o__RF39	0.001024	0	0.3816
g__Defluviitaleaceae_UCG-011	0.001024	0	0.3816
g__norank_f__Coriobacteriales_Incertae_Sedis	0.001024	0	0.3816
g__unclassified_f__Hungateiclostridiaceae	0.001024	0	0.3816
g__Methylobacterium-Methylorubrum	0	0.000519	0.3816
g__norank_f__norank_o__Oscillospirales	0.0005119	0	0.3816
g__Family_XIII_UCG-001	0.0005119	0	0.3816
g__unclassified_o__Oscillospirales	0	0.000519	0.3816
g__UCG-003	0	0.000519	0.3816

Table. S3 The relative abundance of fungal mycobiota in alcohol and control groups at the phylum level.

Species name	Alcohol-mean (%)	Control-mean (%)	P value
p__Ascomycota	97.7	98.96	0.1563
p__Basidiomycota	1.37	0.981	0.2933
p__unclassified_k__Fungi	0.5542	0.0401	0.7459
p__Glomeromycota	0.1506	0	0.1709
p__Mortierellomycota	0.1087	0.01693	0.836
p__Rozellomycota	0.106	0.005346	1
p__Chytridiomycota	0.009801	0	0.3816

Table. S4 The relative abundance of fungal mycobiota in alcohol and control groups at the genus level.

Species name	Alcohol-mean (%)	Control-mean (%)	P value
g__Kazachstania	92.77	98.16	0.03132
g__Eurotium	1.439	0.2771	0.01564
g__Tausonia	0.1996	0.7761	0.634
g__unclassified_k__Fungi	0.5542	0.0401	0.7459
g__Apiotrichum	0.482	0.05257	0.1144
g__Penicillium	0.294	0.07128	0.06588
g__unclassified_f__Dipodascaceae	0.2593	0.0695	0.5244
g__Aspergillus	0.2958	0.03029	0.006809
g__Didymella	0.1586	0.1167	0.2438
g__Epicoccum	0.2272	0	0.1709
g__Cystofilobasidium	0.1898	0.02673	0.1245
g__Fusarium	0.1461	0.02228	0.1035
g__Chaetomium	0.1533	0.006237	0.4066
g__Cladosporium	0.1533	0.005346	0.0245
g__Gibberella	0.1461	0.01069	0.07

g__Talaromyces	0.1399	0.007128	0.006047
g__Nigrospora	0.1381	0.007128	0.2512
g__Entrophospora	0.1452	0	0.3816
g__Neocosmospora	0.1274	0	0.03247
g__Mortierella	0.1087	0.01693	0.836
g__unclassified_p__Rozellomycota	0.106	0.005346	1
g__unclassified_f__Aspergillaceae	0.06593	0.04366	0.6662
g__Wallemia	0.08019	0.02762	0.487
g__Alternaria	0.08197	0.006237	0.7627
g__Issatchenkia	0.07039	0	0.3816
g__Pseudeurotium	0.06504	0.005346	0.2697
g__Ciboria	0.05881	0	0.3816
g__unclassified_o__Pleosporales	0.05257	0.000891	1
g__unclassified_p__Basidiomycota	0.05168	0	0.07645
g__Plectosphaerella	0.0499	0	0.3816
g__Holtermanniella	0.03742	0.008019	0.5656
g__Archaeorhizomyces	0.04277	0	0.1709
g__unclassified_f__Nectriaceae	0.0392	0.003564	0.4881
g__Saitozyma	0.04188	0	0.1709
g__unclassified_f__Didymellaceae	0.01782	0.02406	0.5897
g__Sarocladium	0.04188	0	0.1709
g__Acrostalagmus	0.03831	0	0.1709
g__Periconia	0.03386	0.000891	1
g__Gibellulopsis	0.03386	0	0.1709
g__Naganishia	0.01515	0.01604	0.4078
g__Filobasidium	0.02495	0	0.3816
g__Solicoccozyma	0.02317	0	0.3816
g__Mycoarthris	0.02228	0	0.3816
g__Rhodotorula	0.01604	0.006237	0.7001
g__Trichoderma	0.02138	0	0.1709
g__Acremonium	0.02049	0	0.3816
g__Thielavia	0.0196	0	0.3816
g__unclassified_o__Hypocreales	0.01782	0	0.3816
g__unclassified_o__Tremellales	0.01782	0	0.1709
g__Vermispora	0.01782	0	0.3816
g__Cheilymenia	0.01782	0	0.3816
g__Cryptococcus_f__Tremellaceae	0.006237	0.01158	0.5897
g__Cordyceps	0.01337	0.003564	0.5897
g__Coprinellus	0.01604	0	0.3816
g__Exophiala	0.01515	0	0.3816
g__Microsphaeropsis	0.01515	0	0.3816
g__unclassified_f__Ceratobasidiaceae	0.01426	0	0.3816
g__Articulospora	0.01426	0	0.3816
g__Zopfiella	0.01426	0	0.3816

g__Clonostachys	0.01426	0	0.3816
g__Mrakia	0.01337	0	0.3816
g__unclassified_f__Lasiosphaeriaceae	0.01337	0	0.3816
g__Flammulina	0.01337	0	0.3816
g__Leucoagaricus	0.01337	0	0.3816
g__Cadophora	0.008019	0.005346	1
g__Papiliotrema	0.005346	0.008019	0.4078
g__Cystobasidium	0.001782	0.01158	0.4881
g__Graphium	0.01069	0.001782	1
g__Stagonosporopsis	0	0.01247	0.3816
g__Stachybotrys	0.01247	0	0.3816
g__unclassified_f__Trichosphaeriaceae	0.01247	0	0.3816
g__Paracylindrocarpon	0.01247	0	0.3816
g__Paraconiothyrium	0.01247	0	0.3816
g__Torula	0.008019	0.003564	0.5897
g__Devriesia	0.01158	0	0.3816
g__Ceratobasidium	0.01158	0	0.3816
g__Bjerkandera	0.01158	0	0.3816
g__unclassified_f__Onygenaceae	0.01158	0	0.3816
g__Kernia	0.006237	0.004455	1
g__Coniochaeta	0.01069	0	0.3816
g__Hannaella	0.01069	0	0.3816
g__unclassified_f__Microascaceae	0.01069	0	0.07645
g__Monosporascus	0.009801	0	0.3816
g__Basipetospora	0	0.009801	0.3816
g__unclassified_p__Chytridiomycota	0.009801	0	0.3816
g__Setophaeosphaeria	0.009801	0	0.3816
g__Vishniacozyma	0	0.009801	0.1709
g__Granulobasidium	0.009801	0	0.1709
g__Trechispora	0.00891	0	0.3816
g__Loweporus	0.00891	0	0.3816
g__Microascus	0.005346	0.003564	1
g__Microglossum	0.00891	0	0.3816
g__Minimelanolocus	0.008019	0	0.3816
g__Coprotus	0.008019	0	0.3816
g__Melanocarpus	0.008019	0	0.3816
g__Barnettozyma	0.008019	0	0.3816
g__Cryptococcus_f__Cryptococcaceae	0.008019	0	0.3816
g__Paraphoma	0.008019	0	0.3816
g__Paraphaeosphaeria	0.008019	0	0.3816
g__Westerdykella	0.008019	0	0.3816
g__Thermomyces	0.005346	0.002673	1
g__Ustilaginoidea	0.007128	0	0.3816
g__Coprinopsis	0.007128	0	0.1709

g__Laetisaria	0.006237	0	0.3816
g__Membranomyces	0	0.006237	0.3816
g__Knufia	0.006237	0	0.3816
g__Fusicolla	0.006237	0	0.3816
g__Diutina	0	0.005346	0.3816
g__Trichosporon	0	0.005346	0.3816
g__Scheffersomyces	0.005346	0	0.3816
g__unclassified_f__Saccharomycetaceae	0.005346	0	0.3816
g__Phanerochaete	0.001782	0.003564	0.5897
g__unclassified_f__Sporormiaceae	0	0.005346	0.3816
g__unclassified_f__Phallaceae	0.005346	0	0.3816
g__Saccharomyces	0.005346	0	0.3816
g__Phaeosphaeria	0.005346	0	0.3816
g__unclassified_o__Glomerales	0.005346	0	0.3816
g__Myrmecridium	0.005346	0	0.3816
g__unclassified_f__Plectosphaerellaceae	0.005346	0	0.3816
g__unclassified_c__Sordariomycetes	0	0.005346	0.3816
g__Psathyrella	0.004455	0	0.3816
g__Acanthophysellum	0	0.004455	0.3816
g__Sistotremastrum	0	0.004455	0.3816
g__Pseudopithomyces	0.004455	0	0.1709
g__Karstenula	0.004455	0	0.3816
g__Cyberlindnera	0.004455	0	0.3816
g__Coniophora	0.004455	0	0.3816
g__unclassified_o__Chaetothyriales	0.004455	0	0.3816
g__Arthrographis	0	0.004455	0.3816
g__Acrodontium	0.004455	0	0.3816
g__Eupenidiella	0.003564	0	0.3816
g__Metarhizium	0.003564	0	0.3816
g__unclassified_f__Hypocreales_fam_Incertae_sedis	0	0.003564	0.3816
g__Tetracladium	0.003564	0	0.3816
g__Chrysosporium	0.003564	0	0.3816
g__Acaulium	0.003564	0	0.3816
g__Paurocotylis	0	0.003564	0.3816
g__Buckleyzyma	0.003564	0	0.3816
g__Neodidymelliopsis	0	0.003564	0.3816
g__Stagonospora	0.003564	0	0.3816
g__Coprinus	0.002673	0	0.3816
g__unclassified_f__Chaetomiaceae	0.002673	0	0.3816
g__Schizophyllum	0	0.002673	0.3816
g__Arthrinium	0.002673	0	0.3816
g__Pyrenochaetopsis	0	0.001782	0.3816
g__Scytalidium	0	0.001782	0.3816
g__unclassified_o__Pezizales	0	0.001782	0.3816

g__Leucosporidium	0.001782	0	0.3816
g__Candida	0.001782	0	0.3816
g__Rhinocladiella	0	0.001782	0.3816
g__unclassified_o__Saccharomycetales	0.000891	0	0.3816
g__Pseudombrophila	0	0.000891	0.3816
g__Clavispora	0.000891	0	0.3816

Table. S5 The differentially expressed metabolites between alcohol and the control group.

Metabolite	M/Z	Rt	VIP	P value	FC (Alcohol/Control)	Regulate
3-(4-hydroxy-3-methoxyphenyl)-N-(4-oxobutyl)prop-2-enimidic acid	264.121548	2.9686	1.012	0.04685	1.0421	↑
Noroxymorphone	270.114531	3.148266667	2.16	1.10E-05	1.1431	↑
3a,6b,7b,12a-Tetrahydroxy-5b-cholanoic acid	442.314019	4.85015	1.5139	0.0003083	1.0613	↑
7alpha,12alpha-Dihydroxy-3-oxochola-1,4-dien-24-oic Acid	403.245566	5.026966667	1.0182	0.0005221	1.0246	↑
Lucidone C	405.260968	5.4818	1.0891	0.02624	1.0384	↑
(Z)-6-Tetradecene-1,3-diyne-5,8-diol	482.327527	5.767816667	2.1156	1.69E-06	1.0897	↑
Dihydroceramide C2	344.31393	8.5198	1.5053	0.01981	1.1194	↑
Diacetone alcohol	134.116946	0.729766667	1.2635	0.03523	1.0723	↑
5-L-Glutamyl-L-alanine	219.096474	1.066066667	1.2415	0.01021	1.0869	↑
2-Oxo-1,2-dihydroquinoline-4-carboxylate	190.049038	2.269916667	1.3253	0.0004132	1.0571	↑
4-Aminophenylalanine	181.096304	2.585583333	1.5392	0.003062	1.0797	↑
Ixocarpanolide	514.320862	2.946016667	1.1228	0.03172	1.054	↑
[(16R)-5,7,10-trihydroxy-8,8,10,12,16-pentamethyl-3-[1-(2-methyl-1,3-thiazol-4-yl)prop-1-en-2-yl]-9-oxo-17-oxa-4-azabicyclo[14.1.0]heptadec-4-en-11-yl]oxidanesulfonic acid	625.221889	3.2157	1.8297	0.001638	1.1637	↑
Pinocembrin	257.079241	4.037183333	1.0023	0.02225	1.0692	↑
Gamabufogenin	403.245673	4.3033	1.4548	6.90E-07	1.051	↑
(3alpha,5beta,11beta,17beta)-9-Fluoro-17-methylandrostane-3,11,17-triol	341.245588	4.916083333	1.6115	1.04E-06	1.0568	↑
N(6)-(Octanoyl)lysine	562.454742	5.4818	2.3597	0.001183	1.1844	↑
Pantothenol	428.295635	5.626366667	1.0179	0.006524	1.0264	↑

12b-Hydroxy-5b-cholanoic acid	341.281908	5.74805	2.1863	2.17E-08	1.1106	↑
(3beta,5alpha,6beta,22E,24R)-23-Methylergosta-7,22-diene-3,5,6-triol	445.365197	8.724733333	1.1168	0.001925	1.0284	↑
4alpha-Carboxy-5alpha-cholesta-8-en-3beta-ol	431.34927	10.01711667	1.1482	7.86E-05	1.0306	↑
1alpha,25-dihydroxy-26,27-dimethyl-20,21-methano-23-oxavitamin D3	459.344584	9.108483333	1.2782	0.006673	1.0459	↑
(R)-3,4-Dihydro-2-methyl-2-(4,8,12-trimethyl-3,7,11-tridecatrienyl)-2H-1-benzopyran-6-ol	446.306492	6.7317	2.1385	0.0001321	1.1216	↑
PC(15:1(9Z)/0:0)	480.311474	5.74805	2.6899	2.54E-05	1.1608	↑
3a,7b,21-Trihydroxy-5b-cholanoic acid	442.313704	5.500866667	1.9127	1.65E-06	1.0953	↑
Coprocholic acid	468.365621	5.417	1.2884	3.55E-05	1.0352	↑
3-Isochromanone	149.058937	4.3033	1.0295	0.02832	1.0617	↑
Germacrenone	256.225639	4.170016667	2.7347	3.10E-10	1.2147	↑
6beta-hydroxytestosterone	305.209171	4.12545	1.1454	8.35E-06	1.037	↑
(+/-)-Hexanoylcarnitine	260.184145	2.833816667	1.7687	0.0006057	1.2426	↑
3-Sialyl-N-acetyllactosamine	657.231519	2.518	2.1021	0.01019	1.4993	↑
4-Hydroxyphenylacetylglutamic acid	246.078484	1.877416667	1.122	0.006047	1.0556	↑
6-Acetamido-3-oxohexanoate	188.090977	1.748016667	1.0778	0.007865	1.0431	↑
Noradrenochrome	198.075353	1.748016667	1.2074	0.001165	1.0441	↑
(3S,4S)-3-hydroxytetradecane-1,3,4-tricarboxylic acid	347.209352	1.68315	1.3751	0.03131	1.0984	↑
Hydroxyprolyl-Histidine	251.112546	1.6	1.9355	3.46E-07	1.1117	↑
5'-Deoxyadenosine	252.107918	1.537483333	1.5048	0.02813	1.1033	↑
Adenine	136.061303	1.537483333	1.8529	0.002929	1.1078	↑
Lysyl-Serine	256.127864	1.217066667	2.5688	1.95E-09	1.1995	↑
N-Acetylputrescine	131.117349	0.768233333	1.7935	0.009772	1.1559	↑
(E)-2-Methyl-2-buten-1-ol O-beta-D-Glucopyranoside	266.158477	0.60915	1.2418	0.02787	1.1038	↑

Urothion	324.022285	1.963516667	1.1161	0.0001032	1.0544	↑
Glucosyl (2E,6E,10x)-10,11-dihydroxy-2,6-farnesadienoate	467.209908	3.602383333	2.2812	3.26E-06	1.245	↑
11'-Carboxy-alpha-chromanol	417.300252	8.485366667	1.2027	0.01057	1.0618	↑
(3beta,22R,23R,24S)-3,22,23-Trihydroxystigmastan-6-one	461.362804	8.619583333	1.023	0.003927	1.0407	↑
5,9-Epidioxy-3-hydroxyergost-7-en-6-one	443.315203	8.862883333	1.2789	0.0005222	1.0621	↑
13'-Carboxy-alpha-tocopherol	459.347073	9.107033333	1.5117	0.0008924	1.0781	↑
DG(15:0/18:4(6Z,9Z,12Z,15Z)/0:0)	573.451435	9.240366667	1.0913	0.04773	1.063	↑
DG(15:0/18:3(6Z,9Z,12Z)/0:0)	575.467152	9.28435	1.192	0.02958	1.0716	↑
DG(15:0/18:2(9Z,12Z)/0:0)	577.482611	9.587466667	1.2024	0.03823	1.083	↑
5-L-Glutamyl-taurine	253.04922	0.693133333	1.3244	0.04658	1.1833	↑
Myricatomentoside II	555.186032	3.8242	1.7571	0.03	1.3377	↑
6-Hydroxy-2-bornanone glucoside	375.165203	4.088966667	1.2147	0.01305	1.1923	↑
Frangulanine	481.316447	4.481766667	1.3145	1.54E-05	1.061	↑
20-Hydroxyeicosatetraenoic acid	365.232425	4.7189	1.2178	0.009474	1.0589	↑
MG(20:4(8Z,11Z,14Z,17Z)/0:0/0:0)	423.274519	4.849266667	1.5136	1.52E-06	1.0667	↑
Kinetensin 4-7	592.260654	4.980016667	2.003	6.72E-05	1.1564	↑
Yucalexin P17	363.216981	5.133766667	1.5093	0.001208	1.0912	↑
Ganoderic acid N	529.282786	5.4189	1.4001	1.35E-06	1.0554	↑
16-Acetylpriverogenin A	535.338415	5.4407	1.9757	0.01055	1.3644	↑
(-)-Fumigaclavine B	301.155183	5.569333333	1.0196	0.02995	1.0642	↑
1b,3a,7b-Trihydroxy-5b-cholanoic acid	405.263751	5.67875	1.2947	0.007791	1.0694	↑
Gymnodimine	552.335411	8.419466667	1.5424	0.001381	1.0904	↑
5,6-dehydro Arachidonic Acid	301.216499	8.4635	1.0673	7.33E-05	1.0549	↑
Vitamin D2 3-glucuronide	553.353432	9.0401	1.8948	2.71E-06	1.1458	↑
6-pentadecyl Salicylic Acid	347.258321	9.17395	1.2145	0.0003818	1.0764	↑
Theasapogenol E	525.324103	9.217916667	1.2666	0.0002207	1.0616	↑

2(R)-hydroxydocosanoic acid	355.32098	9.850116667	1.7726	8.83E-05	1.1261	↑
Vitamin D3	429.336523	9.850116667	2.5439	0.001097	1.4743	↑
PE(16:0/16:0)	736.518007	11.65396667	1.0904	0.03051	1.0602	↑
GPEtn(15:0/15:0)	662.475732	10.26225	1.7842	0.0002227	1.135	↑
Cerebronic acid	383.352256	10.08928333	1.7756	5.70E-05	1.1356	↑
Methyl linoleate	633.508769	9.790216667	1.4005	0.0003496	1.0719	↑
Momordol	421.331686	9.730566667	2.106	0.008191	1.299	↑
3,5,9-Trihydroxyergost-7-en-6-one	427.320816	9.6636	2.2527	0.0006133	1.2569	↑
12-hydroxyicosanoic acid	327.289725	9.55085	1.8862	0.0002407	1.1555	↑
Sonchifolin	355.157483	9.17395	1.1782	0.001004	1.059	↑
25-Hydroxyvitamin D2	457.331135	9.0401	1.3	0.002239	1.0786	↑
12-hydroxyheptadecanoic acid	285.242854	8.619583333	1.7709	0.0007488	1.1184	↑
Desglucocoroloside	485.290359	8.375933333	1.2399	0.01108	1.0938	↑
LysoPC(20:3(5Z,8Z,11Z))	590.345831	7.93035	1.1603	0.005166	1.0804	↑
PE(20:4/0:0)	500.277349	7.751283333	1.3426	0.004539	1.087	↑
Asparagoside B	577.373587	7.487333333	1.901	0.0009987	1.2387	↑
Cynaroside A	489.193799	7.069116667	1.0807	0.01853	1.0736	↑
7alpha-hydroxy-3-oxochol-4-en-24-oic Acid	387.253328	6.824533333	1.3016	0.0003458	1.0731	↑
MG(0:0/20:5(5Z,8Z,11Z,14Z,17Z)/0:0)	421.258637	5.914866667	1.0651	0.0006053	1.04	↑
24,25,26,27-Tetranor-23-oxo-hydroxyvitamin D3	403.24826	5.4846	1.2612	0.0002519	1.0588	↑
2-Arachidonylglycerol	423.274517	5.2213	1.6737	7.25E-08	1.0792	↑
19-Nor-5-androstenediol	321.20626	4.958266667	1.1449	0.009928	1.0752	↑
N-[(3a,5b,7a)-3-hydroxy-24-oxo-7-(sulfooxy)cholan-24-yl]-Glycine	528.262514	4.936666667	1.1708	0.001119	1.0655	↑

6-{[2-(2,4-dihydroxyphenyl)-3-(3,7-dimethylocta-2,6-dien-1-yl)-7-hydroxy-6-(4-hydroxy-3-methylbut-2-en-1-yl)-4-oxo-4H-chromen-5-yl]oxy}-3,4,5-trihydroxyoxane-2-carboxylic acid	703.238259	4.827833333	1.2619	0.0281	1.1055	↑
Phytocassane C	363.217004	4.567816667	1.0161	0.01396	1.0501	↑
20, 22-Dihydrodigoxigenin	437.253549	4.265466667	1.6551	1.21E-05	1.1038	↑
19(R)-hydroxy-PGA2	349.201231	3.624833333	1.4001	5.20E-05	1.0779	↑
Artabsin	269.11359	2.803966667	1.1803	0.04292	1.114	↑
3,8-Dihydroxy-6-methoxy-7(11)-eremophilen-12,8-olide	317.138587	2.62655	1.4087	0.01788	1.1529	↑
3,3'-Dihydroxy-4',5,7-trimethoxyflavan	331.117646	2.44815	1.0709	0.03807	1.1061	↑
Biocytin	393.159424	2.337083333	1.032	0.01458	1.2162	↑
Deoxynivalenol 3-glucoside	503.178122	2.138	1.7658	0.01332	1.4984	↑
Asparaginyl-Valine	212.102887	1.358733333	1.1607	5.76E-06	1.0893	↑
FAPy-adenine	134.04572	1.111383333	2.1863	1.03E-06	1.219	↑
12-Ketodeoxycholic acid	355.260952	5.5221	2.4075	0.0003713	0.9044	↓
Dihydrocoumarin	166.085608	1.748016667	1.1569	0.04899	0.9497	↓
Galactosyl 4-hydroxyproline	316.09924	1.558566667	1.2462	0.02447	0.9513	↓
Guanosine	306.07941	1.537483333	1.6729	0.04639	0.8826	↓
D-Pantethine	555.24871	2.7887	1.4092	0.04717	0.9206	↓
Scorpioidin	277.141877	4.806666667	1.0155	0.003933	0.9658	↓
PC(14:0/0:0)	468.305793	7.3324	1.51	0.0002581	0.9482	↓
13(S)-HOTrE	277.214604	7.544266667	1.0236	0.01048	0.9737	↓
LysoPE(16:1(9Z)/0:0)	452.27472	7.587566667	1.8516	0.0003269	0.9105	↓
AMINOHYDROXYBUTYRIC ACID	120.065102	0.710583333	1.2648	0.01532	0.9416	↓
Formylisoglutamine	175.070603	0.729766667	1.2881	0.005905	0.9479	↓
1-Kestose	527.155844	0.749033333	1.0202	0.03801	0.9657	↓
THTC	133.031244	1.152283333	1.6814	0.01036	0.9134	↓

Isovalerylglutamic acid	196.095981	2.76615	1.1868	0.002652	0.9515	↓
Zanthodioline	652.291841	2.585583333	1.542	0.02926	0.8842	↓
5,8,11-Heptadecatriynoic acid	259.167658	5.5221	1.6371	0.0001819	0.938	↓
Neuromedin B (1-3)	303.168677	5.563683333	1.1279	0.01722	0.9603	↓
Thymine	127.049786	1.726466667	1.3089	0.03246	0.9328	↓
D-Pipecolic acid	130.08574	0.786466667	1.6096	0.0001919	0.9427	↓
Hypoxanthine	137.045189	1.415183333	1.0573	0.0191	0.9693	↓
4-Hydroxybenzaldehyde	123.043665	1.500233333	1.3993	0.02255	0.927	↓
5'-Deoxy-5'-(methylthio)adenosine	298.095472	1.832583333	1.1156	0.02306	0.9525	↓
(+/-)-2-(2-Furanyl)pyrrolidine	138.090758	1.899233333	1.1348	0.0005414	0.9614	↓
Petasinine	303.168496	5.86975	1.2576	0.006806	0.9562	↓
3,5-Dihydroxyergosta-7,22-dien-6-one	446.360605	9.28685	3.208	1.50E-06	0.7424	↓
20,24-Epoxy-25,26-dihydroxydammaran-3-one	439.359368	7.289416667	1.4944	0.0005379	0.9415	↓
Porrigenin A	466.349631	6.311116667	1.826	5.51E-07	0.9391	↓
7,8-Dehydro-beta-micropteroxanthin	395.292496	6.311116667	1.8208	9.60E-08	0.9325	↓
3a,7a,12b-Trihydroxy-5b-cholanoic acid	426.319164	5.727333333	1.7752	0.001005	0.9276	↓
Sirolimus	914.559761	5.5221	3.9181	0.0003434	0.6739	↓
Jasmolone	145.10037	5.5221	1.6252	0.0005182	0.9304	↓
Farnesoic acid	237.183425	5.3315	1.2181	0.0004323	0.9584	↓
Prostaglandin E2 ethanolamide	428.297878	5.179733333	1.359	2.97E-07	0.9621	↓
Mauritine A	608.340524	4.192283333	1.3462	0.04178	0.9112	↓
Physapruin B	641.314391	3.949133333	1.4927	0.00381	0.9333	↓
Deoxycholic acid 3-glucuronide	613.296232	3.8612	1.3433	0.03646	0.9357	↓
Phenylalanyl-Tryptophan	334.152947	3.840133333	1.352	0.009099	0.9204	↓
D-Urobilin	621.324704	3.796083333	1.3326	0.03329	0.9359	↓
Oxindole	134.059425	3.081016667	1.1942	0.01631	0.9488	↓

Tetrahydrocortisone	365.230203	2.946016667	1.5867	1.16E-05	0.9349	↓
Arginyl-Gamma-glutamate	344.204756	2.856116667	1.5211	0.0006779	0.9176	↓
1-(2-Furyl)butan-3-one	103.054146	1.748016667	1.3587	0.03285	0.9224	↓
6-(4-ethyl-3-hydroxyphenoxy)-3,4,5-trihydroxyoxane-2-carboxylic acid	297.099964	1.68315	1.8721	0.02501	0.8468	↓
3,4,5-trihydroxy-6-[3-(4-methoxyphenyl)propoxy]oxane-2-carboxylic acid	307.120736	1.6	1.4329	0.04493	0.9036	↓
Spermine	203.222149	1.500233333	2.4454	0.01024	0.7845	↓
4-Guanidinobutanoic acid	146.091746	1.0877	1.4804	0.008333	0.8974	↓
3-Buten-1-amine	72.0811291	0.914783333	1.3047	0.01875	0.9451	↓
Pyrrolidine	72.0811409	0.530533333	1.2663	0.02241	0.9412	↓
2-Hydroxycinnamic acid	165.053929	1.500233333	1.4261	0.01959	0.937	↓
10,11-dihydro-20-trihydroxy-leukotriene B4	385.222397	6.8463	1.1504	0.02647	0.9211	↓
2-Hydroxydecanedioic acid	217.106981	3.49185	1.7969	2.71E-05	0.8776	↓
Physagulin F	543.262094	5.331466667	1.1708	0.0001012	0.9548	↓
Cibaric acid	305.175131	6.153116667	1.0198	0.01583	0.9415	↓
Chenodeoxycholic acid sulfate	471.241147	6.438333333	1.6709	0.001274	0.9107	↓
24,25-Diacetylvulgaroside	541.283061	6.758983333	1.1465	0.0003646	0.9511	↓
L-Arginine	173.102986	0.587983333	2.2497	0.001202	0.6596	↓
Nepetaside	345.15468	3.624833333	2.0607	0.0002236	0.8053	↓
7-Sulfocholic acid	975.479286	5.52745	3.1121	0.0001408	0.7408	↓
3-Hydroxytetradecanedioic acid	255.159312	5.95755	1.7116	0.0001687	0.8787	↓
LysoPC(18:3(6Z,9Z,12Z))	562.313911	7.29175	1.1216	0.02803	0.898	↓
LysoPC(P-18:0)	552.365599	8.4635	1.9951	0.01956	0.7701	↓
9,10,13-Trihydroxystearic acid	367.227051	8.419466667	1.5416	0.01805	0.8587	↓
(+/-)12,13-DiHOME	313.237655	6.394316667	1.0402	0.01728	0.941	↓

6-Hydroxypentadecanedioic acid	269.175181	6.306566667	1.8322	0.001508	0.8392	↓
Isopentyl beta-D-glucoside	545.277862	5.6997	1.5152	1.26E-05	0.9299	↓
6-[2-carboxy-2-(hydroxymethyl)-2-methylethoxy]-3,4,5-trihydroxyoxane-2-carboxylic acid	309.082084	1.448583333	2.3865	0.004241	0.5018	↓
Cis-Resveratrol 3-sulfate	329.011223	0.757983333	1.8495	0.02662	0.2647	↓
N-Acetylneuraminic acid	290.087627	0.7797	1.5991	0.01093	0.8556	↓

Table. S6 The metabolism pathway enrichment analysis.

Pathway	Total	Hits	Raw *p*	-log (P)	Impact_value
Arginine and proline metabolism	72	4	0.0006	3.2388	0.1266
Arginine biosynthesis	23	1	0.1266	0.8977	0.1053
Purine metabolism	81	3	0.0101	1.9976	0.0342
Cysteine and methionine metabolism	56	1	0.2516	0.5992	0.0330
Pyrimidine metabolism	62	1	0.2685	0.5711	0.0319
Biotin metabolism	23	1	0.1266	0.8977	0.0182
Amino sugar and nucleotide sugar metabolism	107	1	0.3504	0.4554	0.0163
Glutathione metabolism	38	2	0.0203	1.6916	0.0155
Glycerophospholipid metabolism	48	1	0.2266	0.6448	0.0091
Pentose and glucuronate interconversions	53	1	0.2426	0.6151	0.0086
Steroid hormone biosynthesis	89	1	0.3260	0.4867	0.0049
Taurine and hypotaurine metabolism	20	1	0.1121	0.9504	0.0000
Pantothenate and CoA biosynthesis	27	1	0.1450	0.8387	0.0000
D-Arginine and D-ornithine metabolism	9	1	0.0539	1.2680	0.0000
beta-Alanine metabolism	25	1	0.1359	0.8668	0.0000
Aminoacyl-tRNA biosynthesis	52	1	0.2395	0.6207	0.0000
Steroid biosynthesis	57	1	0.2545	0.5942	0.0000
Arachidonic acid metabolism	37	1	0.1869	0.7285	0.0000
