# Supplementary figures and images for: Alcohol-induced gut microbiome dysbiosis enhances the colonization of Klebsiella pneumoniae on the mouse intestinal tract
Source: mSystems. 2024 Feb 12;9(3):e00052-24. doi: 10.1128/msystems.00052-24 (PMC10949497; doi:10.1128/msystems.00052-24)

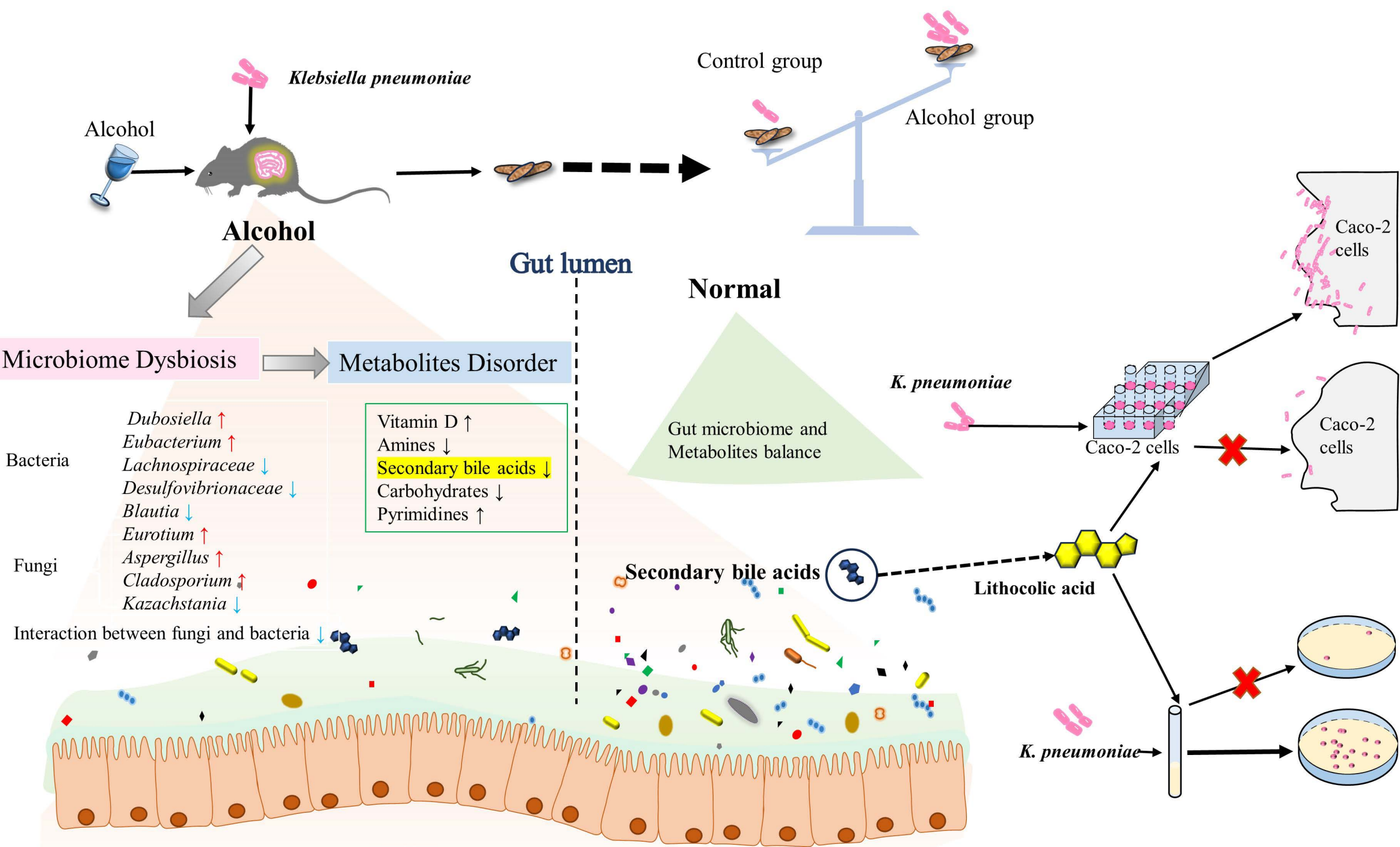

Supplement: Abstract — Graphical abstract. [file msystems.00052-24-s0002.pdf]
